# Whole genome methylation array reveals the down-regulation of *IGFBP6* and *SATB2* by HIV-1

**DOI:** 10.1038/srep10806

**Published:** 2015-06-03

**Authors:** Yinfeng Zhang, Sai-Kam Li, Kevin Yi Yang, Minghua Liu, Nelson Lee, Xian Tang, Hui Wang, Li Liu, Zhiwei Chen, Chiyu Zhang, Jianhua Wang, Stephen Kwok-Wing Tsui

**Affiliations:** 1 School of Biomedical Sciences, The Chinese University of Hong Kong, Hong Kong; 2 Hong Kong Bioinformatics Center, The Chinese University of Hong Kong, Hong Kong; 3 AIDS Institute, The University of Hong Kong, Hong Kong; 4 Institute Pasteur of Shanghai, Chinese Academy of Sciences, Shanghai, China; 5 Centre for Microbial Genomics and Proteomics, The Chinese University of Hong Kong, Hong Kong; 6 Division of Infectious Diseases, Department of Medicine and Therapeutics, Prince of Wales Hospital, The Chinese University of Hong Kong; 7 HKU-AIDS Institute Shenzhen Research Laboratory and AIDS Clinical Research Laboratory, Shenzhen Key Laboratory of Infection and Immunity, Shenzhen Third People’s Hospital, Shenzhen, China

## Abstract

Nowadays, the knowledge in DNA methylation-mediated gene regulation has shed light on the understanding of virus-host interplay in the context of genome alteration. It has also been shown that HIV is able to change the DNA methylation pattern by DNA methyltransferases and such changes can be correlated with the progression of AIDS. In this study, we have investigated the relationship between genome-wide DNA methylation pattern and HIV infection using the methylated DNA immunoprecipitation - microarray method. A pair of monozygotic twins was recruited: one of the twins was infected with HIV while the other was not. Based on data from the microarray experiment, 4679 differentially methylated regions in the HIV positive subject with the significant peak values were identified. Selected genes were then validated in human T lymphocyte CEM*174 cell line and HIV/AIDS patients by comparing with normal subjects. We found that *IGFBP6* and *SATB2* were significantly down-regulated in HIV-infected CEM*174 cells and 3 different cohorts of HIV/AIDS patients while their promoters were predominantly hyper-methylated compared with normal controls. This study also provides a resource for the identification of HIV-induced methylation and contributes to better understanding of the development of HIV/AIDS.

After more than 30 years of HIV discovery, the pandemic acquired immunodeficiency syndrome (AIDS) has killed more than 35 million people worldwide. Despite the tremendous progress made in various aspects of the virus and its interplay with the host, the mechanisms underlying HIV pathogenesis remains poorly understood.

It is known that endogenous retroviruses in the human genome can be silenced epigenetically by the host DNA methyltransferases (DNMTs), including transcriptional repression of the proviral DNA, modifying the chromatin conformation, and epigenetically methylating the DNA at the enhancer/modulatory region^1^. Such hyper-methylation can also happen to the integrated HIV genome, which may explain the virus latency in chronic infections. In detail, the HIV long terminal repeats (LTR) was hyper-methylated at the CpG sites in the 5’-LTR but not the 3’-LTR. Moreover, De-methylation of CREB/ATF sites in the LTR has also been shown as a crucial step for the reactivation of latent HIV-1 genome *in vivo*^2^,^3^. In contrast, many eukaryotic viruses can interact with host cellular factors that contribute to the alteration of DNA methylation and eventually the downstream host gene transcription.

It is well known that aberrant DNA methylation in cancer cell genomes may attribute to the effect of cancer-inducing viruses^4^. Intriguingly, several viral oncogenic proteins were found to be the interacting partners of cellular DNMTs and histone-modifying enzymes. Basically, mammalian CpG methylation is carried out by three active DNA methyltransferases: DNMT1, DNMT3A, and DNMT3B^5^. DNMT1 has a higher preference for hemi-methylated DNA as the substrate. DNMT3A and DNMT3B are mainly regarded as *de novo* DNA methyltransferases, whose roles are critical in the dynamic DNA methylation process during embryogenesis and pathogenesis. However, it is also suggested that DNA methyltransferases do not work solely in maintenance or *de novo* methylation^6^,^7^,^8^.

Although HIV-1 is not regarded as a tumor virus, it was reported that HIV-1 triggers hyper-methylation of some host genes, e.g. HIV-1 infection of lymphoid cells could lead to the increase in DNMTs expression^9^,^10^,^11^, as well as the hyper-methylation and reduced expression of FOXP3^10^, IFN-γ and p16INK4A^12^. In contrast to the myriad studies of HIV-1-induced differential gene expression, the genome-wide alterations in DNA methylation induced by HIV-1 infection have not been described. To provide novel insights to HIV/AIDS pathogenesis, we identified the alterations of host genome methylation driven by HIV-1 using high-throughput technologies in this study. The effect of methylation on expression of selected genes was then validated at both transcriptional and translational levels. In summary, our results contribute to a comprehensive picture of epigenetic regulation of genes by HIV-1 in T cells.

## Results

### Diagnosis of HIV infection

A pair of 15-year-old female identical twins was recruited for this study. More clinical information of the twins could be found in the Materials and Methods section. The result of the fast screen test and Western blot confirmation showed that one of the twins was infected with HIV-1 while the other was not (Supplementary Figure. S1a and S1b). The CD3+, CD4+ and CD8+ cell counts were given in Supplementary Table S1.

### Confirmation of the zygosity of the twins

To validate the monozygosity of the twins, matches for short tandem repeats (STRs) were determined. Referring to the Supplementary Table S2, no genetic discrepancy between the HIV positive and negative subjects was observed in any of the 15 STR loci. Identical patterns in the two DNA samples at 15 STR markers were also shown in Supplementary Figure. S2. These results provided solid evidence to confirm the monozygosity of the twins.

### Genes differentially methylated in the HIV+/- twins

Since the samples from HIV positive and negative subjects were hybridized onto the same chip, generated methylation peaks could be considered as unique peaks to one of the subjects, or designated as Differentially Methylated Regions (DMRs) if the peak value is above 3.0. Generally, two types of DMRs were identified: (1) methylated only in HIV+ twin and (2) methylated only in HIV- twin. In an effort to filter out false positives with an in-house script, if the methylation peak from either of the twins could be annotated to the same gene or region, the peak would be considered as the overlapped peak and therefore deleted from the DMRs list.

**Figure 1:**
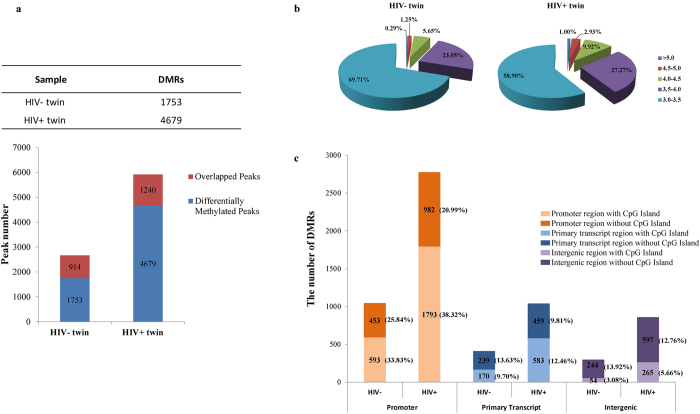
Data analysis of methylation microarray. (**a**) The differential methylation regions (DMRs) in both twins. The red bar indicated the overlapped methylation regions while the blue bar indicated the differentially methylation regions. A total of 4679 DMRs were found in HIV positive twin and 1753 DMRs in the HIV negative twin. (**b**) Numbers of high value DMRs in the genome-wide DNA methylation profiles in both twins at different value range. It was assumed that high value DMRs have the peak values over 3.0. (**c**) Numbers of DMRs at different distance range from transcription start sites in both twins. DMRs were divided into 3 categories according to their location from the genes they were mapped to. Numbers in the parentheses indicate the percentage.

Using the NimbleGen array data analysis software, a total of 5919 and 2667 methylation peaks were identified in the HIV+ twin and HIV- twin, respectively. After filtering the overlapped peaks as mentioned above, there were a significantly larger number of DMRs unique to HIV+ twin (4679) and a significantly smaller number unique to HIV- twin (1753) (χ^2^ test, *P* < 0.001, Fig. 1a).

In addition, by comparing the percentage of DMRs at different value ranges, we found another characteristic feature of the genomic DNA methylation profiles between the twins. The result showed the significantly increased proportion of high methylation peaks (with the values above 3.5) in HIV+ twin than HIV– twin (χ^2^ test, *P* < 0.001, Fig. 1b), especially at the ranges of 4.0-4.5, 4.5-5.0 and above 5.0, the DMR proportion of HIV+ twin were nearly twice as high as that in her normal sibling. In order to determine the proportion of DMRs in different genomic regions, the numbers of DMRs were compared in the promoter (defined as 1000 bp from the TSS), primary transcript and intergenic regions according to the rules mentioned in the section of Materials and Methods based on the Nimblegen microarray design. In both twins, >50% of the DMRs were located in promoter regions (Fig. 1c). Moreover, DMRs in promoter regions were higher in HIV+ twin while DMRs in other genomic regions of both subjects were similar.

As predicted by the software of Idiographica V 2.1^13^, several chromosomal loci displayed positional enrichments of DMRs in HIV+ twin by considering the length of chromosome, such as chromosome 17, 19 and 22 (Supplementary Figure. S3).

### Functional profiling of genes differentially methylated in HIV+/- twins

To determine whether there were any common categories in the identified methylated genes, we performed the gene ontology functional classification. Considering that the distance of methylated CpG islands from transcription start sites would affect the gene expression regulation^14^,^15^, we divided all DMR-associated genes with peak value >3.0 based on their genomic locations of DMRs into three different groups - non-CpGi promoter, CpGi promoter and primary transcript. As shown in Table 1, biological process, molecular function and cellular component categories are significant GO terms in the CpGi promoter group in HIV+ twin. Within the category of biological process, the most significant functional terms were “regulation of transcription” in the HIV+ twin and “regulation of neuron apoptosis” in the HIV- twin. All statistically significant functional GO categories (*P* < 0.001) in other groups are listed in Supplementary Table S3. When KEGG pathway analysis was used to further analyze DMRs in CpGi-promoter region of HIV+ twin, pathways involved in ribosome, pyrimidine metabolism and steroid biosynthesis were significantly enriched (*P* < 0.05) (Table 1).

### Validation of the MeDIP/ microarray results in human CEM*174 T lymphocyte cell line and HIV/AIDS patients

**Figure 2:**
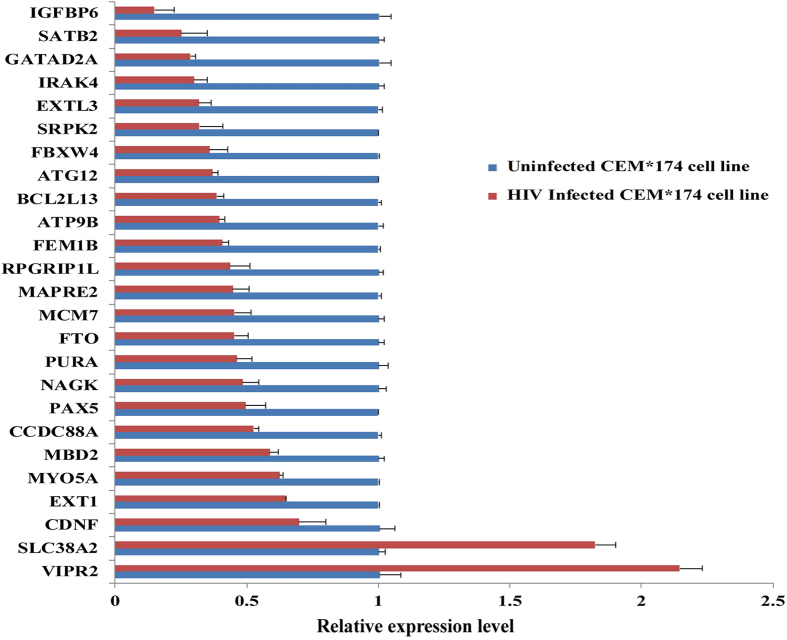
Relative transcript level of 25 genes in CEM*174 T cell line before and after HIV infection. The mRNA expression level of selected genes were determined and normalized with β-actin mRNA. Results were given as fold change in mRNA expression with ± SD. Independent experiments were repeated three times.

Next, we selected 25 hyper-methylated target genes in HIV+ twin based on their peak values and results of GO classification for validation in T lymphocyte cell line by real-time PCR. Our results showed that the transcriptional level of most of the target genes (18/25) were decreased by more than 50% in the HIV+ twin (Fig. 2). In particular, some genes exhibited a bigger decrease in expression level, such as *IGFBP6* (6.72 fold) and *SATB2* (3.99 fold).

**Figure 3:**
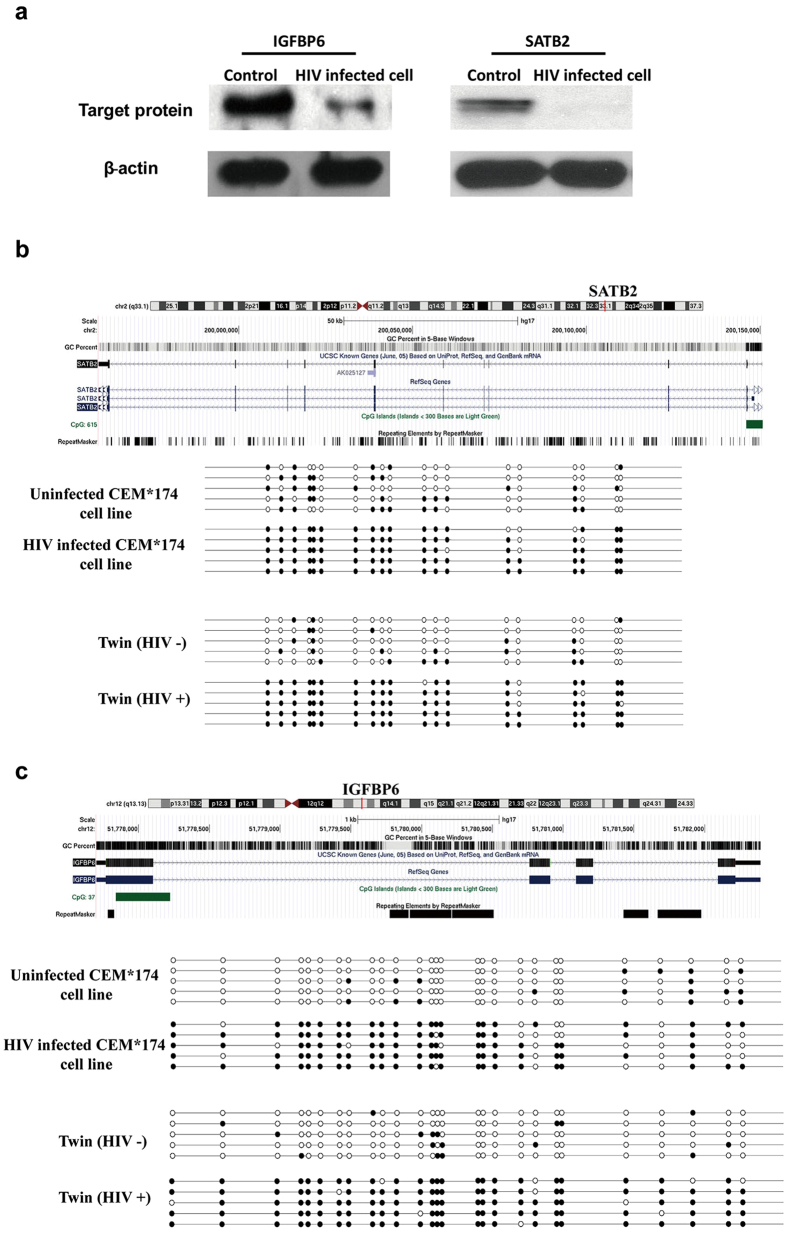
The expression level and methylation status of 2 target genes in CEM*174 T cells. (**a**) Western blotting result of IGFBP6 and SATB2 in HIV infected/uninfected CEM*174 cell lines. Full-length blotting images are presented in Supplementary Figures. The bisulfite sequencing result of (**b**) *SATB2*, (**c**) *IGFBP6* in HIV infected/uninfected CEM*174 cell lines and both twins were shown. Solid circle represented methylated CpG islands; Open circle represented unmethylated CpG islands.

For further verification, *IGFBP6* and *SATB2* from the 25 genes were selected because of their high peak values (*SATB2* = 5.55, *IGFBP6* = 5.08), greater fold changes at the transcriptional level and the potential relationship with HIV infection according to literatures^16^,^17^,^18^. The western blotting result showed that the alterations could also be observed at the protein level. The amounts of both target proteins have been decreased significantly upon the HIV infection (Fig. 3a). The β-actin was used as the internal reference.

Afterwards, we examined the methylation status of the CpGi in promoter regions of *IGFBP6* and *SATB2* in both twins and HIV-infected/uninfected CEM*174 cell lines using bisulfite sequencing. Each row from the Fig. 3b,c represents an individual clone with solid circle representing the methylated CpG site whereas open circle as the unmethylated CpG site. The result suggested that the promoter region of *IGFBP6* and *SATB2* in HIV+ twin was hyper-methylated. This hyper-methylation was absent in the HIV- twin. Consistently, the hyper-methylation pattern could also be observed in the T cell line upon HIV infection.

**Figure 4:**
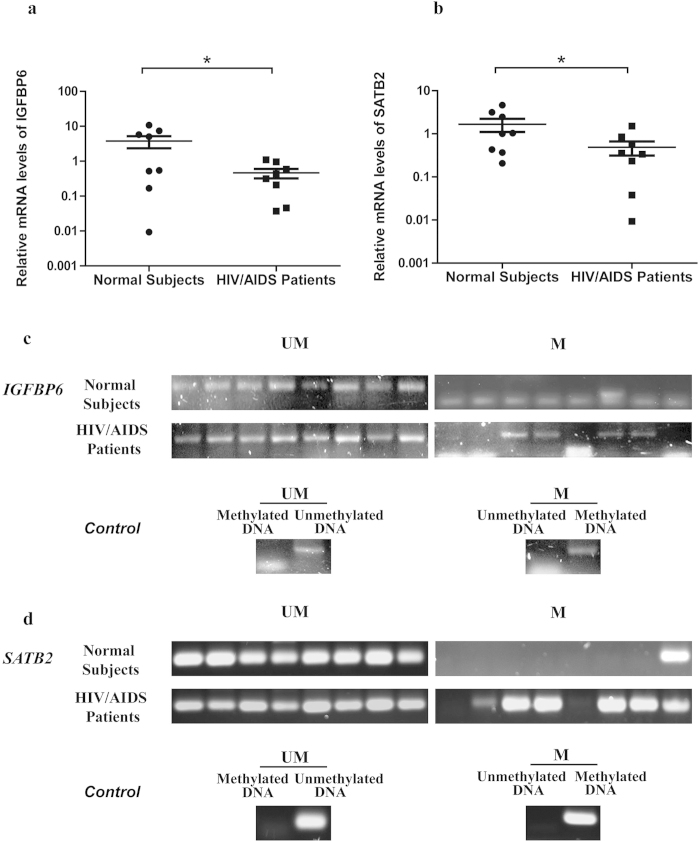
Comparison of relative expression level and methylation status of IGFBP6 and SATB2 between 8 HIV/AIDS patients and normal subjects. The mRNA expression levels of *IGFBP6* (**a**) and *SATB2* (**b**) were analyzed in 8 HIV/AIDS patients and 8 normal people. Round dots represent normal control; Rectangle dots represent HIV/ AIDS patients. The y-axis is plotted on a logarithmic scale. * *P* < 0.05, Mann-Whitney U test. Methylation status of *IGFBP6* (**c**) and *SATB2* (**d**) were checked by MSP. From left to right lanes in the HIV/AIDS patient panel, bands represent the methylated PCR products from patients 1 to 8. M, PCR for CpG-methylated DNA; UM, PCR for CpG-unmethylated DNA. Fully uncut gel images are shown in Supplementary Figures.

In addition, 8 HIV/AIDS patients and 8 normal subjects were recruited to further validate this finding. The result showed that both genes were down-regulated in the group of HIV/AIDS patients at the transcriptional level. After normalization by the β-actin, the expression level of *IGFBP6* was down-regulated by 65.9% in HIV/AIDS patients relative to normal controls (Fig. 4a, *P* = 0.04, one sided Mann-Whitney U test) while *SATB2* was down-regulated by 57.1% (Fig. 4b, *P* = 0.027). In addition, Fig. 4c,d showed the methylation status of these two genes in both patients and normal subjects. In the promoter region of *IGFBP6*, 4 out of the 8 patients (50%) and none of the normal subjects (0%) were methylated while in that of *SATB2*, the promoter region of 6 patients (75%) and one normal subject (12.5%) were methylated.

**Figure 5:**
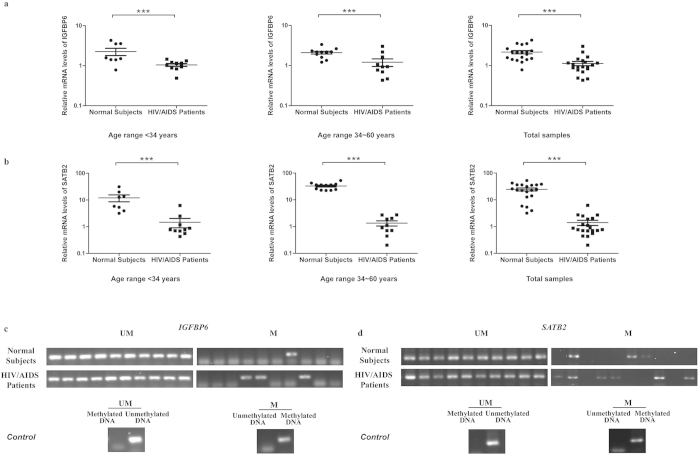
Decreased expression of target genes between 20 HIV/AIDS patients with similar viral load and 20 age- and sex-matched normal subjects. All HIV/AIDS patients did not receive any antiretroviral therapy. The mRNA expression levels of IGFBP6 (**a**) and SATB2 (**b**) were analyzed in 3 groups: age under 34 years (8 normal subjects vs 12 HIV/AIDS patients), age between 34 and 60 years (12 normal subjects vs 8 HIV/AIDS patients) and all samples (20 normal subjects vs 20 HIV/AIDS patients). Round dots represent normal control; Rectangle dots represent HIV/AIDS patients. The y-axis is plotted on a logarithmic scale. *** P < 0.005; ** P < 0.01; * P < 0.05, Mann-Whitney U test. Methylation status of IGFBP6 (**c**) and SATB2 (**d**) were checked by MSP. M, PCR for CpG-methylated DNA; UM, PCR for CpG-unmethylated DNA. Fully uncut gel images are shown in Supplementary Figures.

To reduce the impact caused by other factors such as sex, age and antiretroviral reagents on methylation and differential expression, we have further recruited 20 HIV infected male patients with similar viral load who did not receive any antiretroviral therapy and 20 normal subjects with age and sex matched (The clinical information was shown in Supplementary Table S6). According to the distribution of ages, samples have been divided into two groups: age under 34 years (8 normal subjects vs 12 HIV/AIDS patients) and age between 34 and 60 years (12 normal subjects vs 8 HIV/AIDS patients). Consistent with our previous results, we found that the relative expression levels of both *IGFBP6* and *SATB2* were low in all groups of HIV/AIDS patients compared with normal groups. Normalized by the β-actin, in the group with “ age under 34 years”, “age between 34 and 60” and “all samples”, the expression level of *IGFBP6* was down-regulated by 53.7%, 42.4% and 47.8%, respectively, in HIV/AIDS patients relative to normal controls (Fig. 5a, P < 0.001, two sided Mann-Whitney U test) while *SATB2* was down-regulated by 87.8%, 95.9% and 94.3%, respectively (Fig. 5b, P < 0.001). Furthermore, MSP experiment were also performed in HIV infected patients with age between 34 and 60 years. Due to the limitation of samples, another batch of 10 male normal subjects with age matched has been recruited for the comparison. Figure 5c,d showed the methylation status of these two genes in both patients and normal subjects. In the promoter region of *IGFBP6*, 3 out of the 10 patients (30%) and 1 out of the normal subjects (10%) were methylated while in that of *SATB2*, the promoter region of 6 patients (60%) and 3 normal subject (30%) were methylated. Therefore, our results indicated that HIV infection was involved in the differential methylation and transcriptional regulation of *IGFBP6* and *SATB2*.

**Figure 6:**
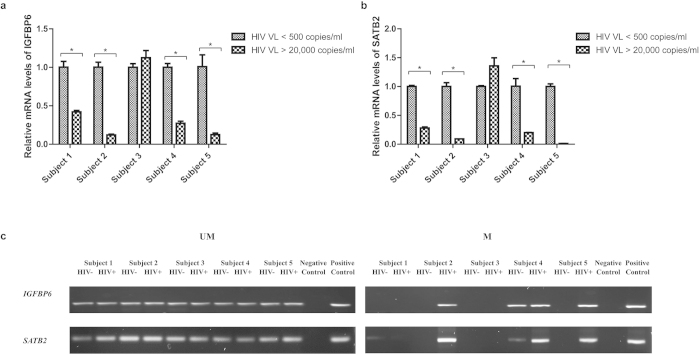
The comparison of expression level and methylation status of target genes in HIV infected patients with high and low viral load. The mRNA expression levels of *IGFBP6* (**a**) and *SATB2* (**b**) were analyzed in 5 HIV infected subjects with different viral load. * *P* < 0.05, Paired Student T test. Data represent means ±SD from at least 2 independent experiments. HIV VL < 500 indicated that the sample was collected when the viral load of the subject was lower than 500 copies/ml; HIV VL > 20,000 indicated the viral load was higher than 20,000 copies/ml. Methylation status of *IGFBP6* and *SATB2* (**c**) were checked by MSP. M, PCR for CpG-methylated DNA; UM, PCR for CpG-unmethylated DNA. If the viral load of the sample was lower than 500 copies/ml or higher than 20,000 copies/ml, it would be termed as “HIV −”or “HIV +”, respectively. Fully uncut gel images are shown in Supplementary Figures.

To further validate the above observation, we have recruited 5 HIV infected subjects with multiple visits. For each patient, a blood sample with a very high viral load (>20,000 copies/ml) was collected during the first visit and then another sample with a very low viral load (<500 copies/ml) was taken during the second visit (Supplementary Table S6). Since these paired samples were collected from the same subject, the results would reflect the relationship between the viral load and our target genes. We found that 80% (4 out of 5) of samples with high viral load had a lower expression of *IGFBP6* and *SATB2* when compared to samples with low viral load (Fig. 6a,b). Meanwhile, the MSP experiment showed that 3 out of 5 subjects for the *SATB2* and 2 out of 5 subjects for *IGFBP6* were consistent with our previous findings (Fig. 6c).

### Determination of DNMTs expression in HIV infected cells

**Figure 7:**
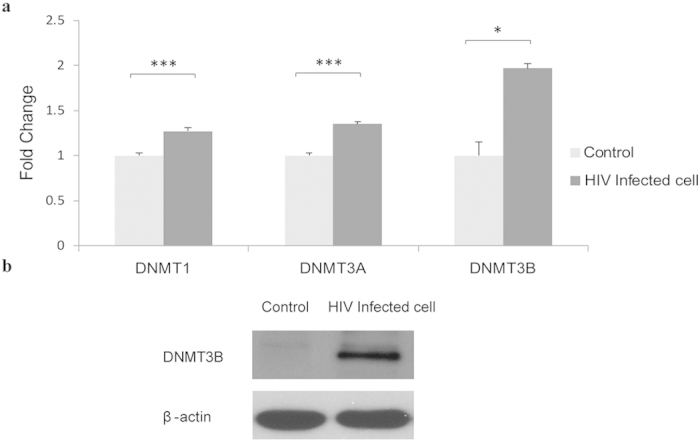
Determination of DNMTs expression. (**a**) Relative transcript levels of DNMT1, DNMT3A and DNMT3B (*** *P* < 0.005; ** *P* < 0.01; * *P* < 0.05; Paired Student T test) and (**b**) Western blot result of DNMT3B in HIV infected and uninfected CEM*174 cell lines. Data represent means ±SD from 3 independent experiments. Full-length blotting images are presented in Supplementary Figures.

It has been reported that HIV can change the expression of DNMTs, resulting in the alterations of DNA methylation across the genome. Therefore, we have also checked the differential expression of DNMT1, DNMT3A and DNMT3B in CEM*174 T cell line during HIV infection. The results showed that after viral infection, both DNMT1 and DNMT3A displayed a significant increase in expression (*P* < 0.005). However, DNMT3B showed a relatively higher expression (*P* = 0.01) (Fig. 7a). Therefore, the DNMT3B was chosen for further analysis. As expected, the western blotting data confirmed the real-time PCR results - the protein level of DNMT3B was greatly increased in HIV-infected cells than the control (Fig. 7b), which indicated that the HIV may affect DNMT3B and up-regulate its expression to change the DNA methylation pattern across the genome.

## Discussion

Nowadays, the genome-wide DNA methylation study was greatly facilitated by the use of deep sequencing or gene tiling arrays. However, there was very limited number of studies on DNA methylation induced by HIV in contrast to the importance of HIV in scientific/biomedical research. One major obstacle was the lack of an appropriate model. It has been well known that the epigenetic alteration is regulated by both genetic and environmental factors, which mean individual variations are enormous among individuals^19^,^20^. Therefore, finding an ideal model with individuals having nearly identical genetic background would be very important to HIV methylation research. Due to the ethical consideration and practical situation, it is difficult to obtain samples from the same person before and after HIV infection. One of the solutions is the use of SIV in non-human primate model. However, because of the genetic variations between SIV and HIV as well as the different epigenetic regulation patterns in human and monkey, the results generated could not totally reflect the real situation in HIV patients. In this study, the solution we explored is the model of identical twins. Twins have been commonly recruited in scientific research because of the perfectly matched genetic background and very similar living environment^21^,^22^. Furthermore, in the context of epigenetic variations, it has been shown that monozygotic twins have similar methylation content in their genome, in contrast to that of dizygotic twins and other unrelated persons generally^23^,^24^,^25^. Therefore, by comparing the epigenetic regulation between monozygotic twins, the genetic influences on epigenetic changes are eliminated while the variations could be attributed to exogenous factors such as viral infection. In this study, comparing to her healthy sibling and the reference range of healthy population from 3 different districts in China: Beijing, Hong Kong and Shanghai^26^,^27^,^28^, the HIV+ twin had a significantly reduced count of CD4+ cells of <350 cells/microliter (Supplementary Table S1) and an abnormal CD4/CD8 cell ratio, suggesting that the HIV+ subject was at the moderate advanced stage of AIDS. The model is also reliable because the HIV+ twin has not received any antiretroviral therapies, meaning that there were persistent interactions between the virus and the host as well as reduced number of confounding factors on methylation.

In our study, the MeDIP method followed by promoter CpGi microarray analysis was utilized to identify DMRs. Since this is a pioneer study without previous references, more conservative criteria were applied to identify and eliminate overlapped methylated DMRs. Although the total number of DMRs may be underestimated because DMRs with low CpG density could be missed by MeDIP^29^, almost 5000 DMRs in HIV+ twin and 1800 DMRs in HIV– twin were identified. Due to the limited amount of blood samples, the PBMCs were analyzed and this practice is common in HIV research. To justify the use of PBMCs, we have overlapped the detected DMRs with loci exhibiting CD4 T cell-specific methylation using a bioinformatics approach together with literature search. Briefly, 601 from 1887 genes differentially methylated in CD4 T cells^30^,^31^,^32^,^33^,^34^,^35^,^36^,^37^,^38^ were overlapped with our databases of DMRs. Our results showed that 395 genes and 206 genes were found in the lists of hyper-methylated DMRs from the HIV positive twin and HIV negative twin, respectively. This implies that 8.44% and 11.75% of DMRs in the HIV+ twin and HIV- twin are CD4 T cell type specific (Supplementary Table S7). Moreover, to estimate the proportion of DMRs associated with HIV infection in our database, we have overlapped the detected DMRs with reported loci exhibiting different methylation in PBMCs of other MZ twins without HIV infection using a bioinformatics approach together with literature search. Our results showed that 182 (142 genes in HIV+ twin and 40 genes in HIV- twin) out of the 1458 genes in our databases of DMRs are differentially methylated in MZ twin pairs^23^,^39^,^40^,^41^,^42^,^43^,^44^ in the literature. This implied that only 3.0% and 2.3% of DMRs in our HIV+ twin and HIV- twin were overlapped with DMRs known to be variable in MZ twin pairs (Supplementary Table S7). Based on the results, we have the confidence that authentic and significant number of DMRs from the MZ twins associated with HIV infection could be captured in this study for validation in subsequent experiments.

Moreover, our results showed that many more methylated DMRs as well as higher average peak values were found in HIV+ twin when compare with HIV- twin, implying that the HIV infection would cause the increase of global methylation level. This finding is consistent with several reports mentioned that HIV infection triggers the up-regulation of DNA methyltransferases. For example, an up-regulation of DNMT1 could be triggered by the expression of non-structural HIV genes, including nef, tat and rev, in an *in vitro* HIV acute infection model^9^,^11^. In addition, up-regulation of DNMT3B upon HIV infection has been previously demonstrated^10^,^45^. It is notable that similar phenomenon has also been observed in our study, although the detailed mechanism deserves further investigation. Another interesting result is the chromosomal locations of DMRs in HIV+ twin. According to our bioinformatics analysis, positional enrichments of DMRs were located in chromosomes 17, 19 and 22. It has been proposed that HIV prefers to integrate into actively transcribed genes for efficient expression of viral genome rather than integrating randomly^46^. All three chromosomes with high gene density have been reported to be preferred for HIV integration^47^,^48^. Therefore, one plausible explanation of the phenomenon was that the host utilizes the DNA methylation to suppress the HIV propagation.

In both subjects, >50% DMRs were located in promoter regions while more than 30% of them were located in CpGi promoter regions. It was lower than the proportion of promoter CpGs represented in NimbleGen array partly because of our relatively conservative screening criteria. In addition, because the input and control samples were both methylated DNA fragments captured by antibody from HIV+ and HIV- twins, same methylated regions may not be displayed in the subsequent analysis. When DMRs were divided into different groups according to their genomic location, we found that significant differences could be found in many important biological processes when the CpGi promoter groups of HIV+ and HIV- twins were compared. Moreover, it is interesting that the steroid biosynthesis pathway was identified by KEGG in the DMRs located in CpGi promoter region of HIV+ twin (Table 1) because HIV infection has been reported to contribute to dyslipidaemia^49^ and some steroid hormones, like estrogens, have been described to reduce the HIV infection^50^. Whether the disruption of steroid biosynthesis by DNA methylation was a strategy exploited for HIV replication deserves future investigation.

Although MeDIP-NimbleGen microarray is one of the powerful methods to detect the regional methylation changes at a genome-wide scale, some limitations still exist. One of the concerns is that the MeDIP-NimbleGen microarray method tends to find the methylated regions with CpG rich content. Despite of the fact that the MeDIP-microarray method can provide more precise result in high CpG region^29^, the differentially methylated regions in low CpG regions may be lost. Potential impacts can also be inferred from the complicated correlations between DMRs and their impact on gene expression. One of the situations is the usage of alternative promoter. Since the alternative promoter may be utilized by different splicing isoforms, DMR located in the promoter of one isoform could not exert regulation effect on other isoforms with alternative promoter. Furthermore, multiple DMRs can be correlated with one gene at the same time. All these limitations highlight the importance to critically verify the results from MeDIP-microarray. Therefore, our microarray results were further confirmed in the CD4+ T lymphocyte cell line and recruited HIV/AIDS patients. For validation, the most distinguished genes from the DMRs list with high peak values were focused so as to minimize the possibility that the methylation was caused by individual variations. The results from CEM*174 T cell line showed that most of the selected genes were down-regulated. In addition, we also observed the down-regulation of *IRAK4* which has been previously reported to be methylated during HIV infection^51^. This finding can serve as a positive control of our study. To further verify our result, we selected 2 representative genes with high peak values and greater alterations at the transcriptional levels. One of the reasons for the selection was that a few articles showed their potential association with HIV, although both genes have not been reported to play a role in the process of viral infection. *SATB2* is the modulator of ΔNp63α^16^, which may interact with HIV-1 Tat protein^17^. *IGFPB6* is a member of the IGFBP super-family, whereas the levels of *IGFBP1*, 2 and 3 have been observed to vary in patients with HIV/AIDS^18^. As expected, both target genes were down-regulated at the protein level in T cell line upon HIV infection while the BSP result showed that their promoter regions were hyper-methylated. It was consistent with the fact that the methylation peaks annotated to *IGFBP6* and *SATB2* from the chip were located in promoter regions. The promoter methylation of these two genes was also demonstrated by the BSP result of the twins. Furthermore, combined with the reduced mRNA level and the MSP results in three cohorts of HIV/AIDS patients compared with normal control, our results indicated that HIV infection was involved in the differential methylation and transcriptional regulation of *IGFBP6* and *SATB2*. More importantly, the expression of these two genes were potentially associated with the viral load of HIV. This finding has not been reported before and these two genes may be good targets for future HIV/AIDS research. Taken together, the efficacy of the MeDIP-microarray approach for the identification of genes epigenetically regulated by HIV-1 has been well demonstrated in this study and identified DMRs could be applied to a wide range of HIV/AIDS research.

To our knowledge, this is the first report discussing the genome-wide DNA methylation associated with HIV-1 infection in monozygotic twins. Our findings contribute to the understanding of HIV-associated global methylation and provide a draft of HIV epigenome.

## Methods

### Ethical Statement

The study was approved by the Institutional Ethic Committee of Jiangsu University, China and the Institutional Review Board of the Chinese University of Hong Kong. All experiments were carried out in accordance with the ethical guidelines concerning the use of human research participants approved by the Joint Chinese University of Hong Kong-New Territories East Cluster Clinical Research Ethics Committee (CREC Ref. No: CRE-2012.416). Written informed consent was obtained from all subjects involved in this study for the collection of samples and subsequent analysis.

### Human blood samples

A pair of 15-year-old female identical twins was recruited for further study based on fast screening test utilizing the Colloidal Gold kit and HIV Blot 2.2 Western Blot confirmation assay (Genelabs). The cell-counts were determined by FACS counting using BD Trucount tubes (BD Biosciences). Both twins were high-school students while none of them was drug-user. The infection was a clinical accident when one of the twins was injected with contaminated needle. The HIV positive twin has not received any antiretroviral therapy. Afterwards, the two batches of blood samples from 8 and 20 HIV/AIDS patients were collected from the Prince of Wales Hospital and Shenzhen Third People’s Hospital while the samples of healthy subject were obtained from Hong Kong Red Cross Blood Transfusion Service or Prince of Wales Hospital, respectively. The blood samples from 5 HIV infected patients with high viral load and low viral load were collected from the Shenzhen Third People’s Hospital. The clinical records of normal subjects could not be disclosed because of the privacy protection while all records of HIV/AIDS patients were shown in the Supplementary Table S6. The PBMCs were separated from the EDTA-blood collected from the blood samples. Total genomic DNA was extracted from PBMCs using QIAamp DNA Mini Kit (QIAGEN) according to the manufacturer’s instructions. QIAamp RNA Blood Mini (QIAGEN) kit was used to extract total RNA from the HIV/AIDS patients and normal subjects.

In addition, to verify whether the twins were actually monozygotic, their genomic DNA was analyzed by STR PCR with the Applied Biosystems AmpFlSTR^®^ Identifiler^®^ PCR Amplification Kit examining 15 + 1 loci.

### Immunoprecipitation of cytosine methylated DNA (MeDIP) and amplification of DNA

Immunoprecipitation of methylated DNA was performed using the Methylated-DNA IP Kit (Zymo Research). In order to acquire enough DNA for microarray, the immunoprecipitated methylated-DNA was amplified using GenomiPhi V2 kit (GE Medical Systems)^52^.

### DNA labelling and hybridization

DNA labelling and hybridization was performed according to NimbleGen’s protocol. The immunoprecipitated CpG-methylated DNA from HIV positive subject (test) and from HIV negative subject (input control) were labelled with Cy5 and Cy3. The DNA samples were then co-hybridized onto NimbleGen Human DNA Methylation 2.1M Deluxe Promoter Array. Raw data of methylation microarray were deposited at GEO under accession number GSE68028.

### Data acquisition and processing

The data scanning and collections were performed using MS 200 Microarray Scanner and MS 200 Data Collection Software. Finally, the result of microarray was analyzed by DEVA version 1.2.1 software (Roche, Nimblegen) using default parameters. After the extraction of signal intensity data from the scanned images, the results of log_2_ ratios, P-score, and peaks were processed and obtained. Log_2_ ratios represent the ratios of test signal of immunoprecipitated DNA from HIV positive subject to the input control signal ratio of immunoprecipitated DNA from HIV negative subject co-hybridized to the array. From Log_2_ ratio data, a 750bp fixed-length window was placed around each consecutive probe followed with Kolmogorov-Smirnov (KS) test, and the resulting score called P-score was the -log_10_ *P*-value for each probe. Finally, the methylation peak data was generated from the P score data through merging consecutive probes with the following default conditions: minimum cut-off p-value 2.0, maximum 500 bp spacing and minimum of two probes per peak.

### Microarray data analyzing

The methylation peaks were mapped to features (transcription start sites, primary transcripts, CpG islands, and other tiled regions) using DEVA software v.1.2.1. Furthermore, to categorize the methylation peaks, the location was defined in context of a gene with the annotation data of promoters, CpG island regions, primary transcripts as well as their genome coordinates from NimbleGen based on UCSC 2010 reference genome hg19. Each methylation peak could be classified into six different categories according to their locations using an in-house script: Promoter region with CpG Island; Promoter region without CpG Island; Intergenic region with CpG Island; Intergenic region without CpG Island; Primary transcript region with CpG Island; Primary transcript region without CpG Island. In detail, the promoter region was defined as the 1-kb region flanking each side of the transcription start site (TSS); the intragenic region of primary transcript was defined as the region inside a gene excluding the promoter; the intergenic region was defined as the region 2 kb away from any annotated genes. Depending on whether the methylation peak located in the CpG island region, all three groups were further categorized in with or without CpG island sub-groups.

### Gene function annotation and pathways analyses

Gene functions were annotated using The Database for Annotation, Visualization and Integrated Discovery (DAVID) 53. We have adapted the official symbols for the gene names and annotation sources GO: BP (biological process), CC (cellular component) and MF (molecular function) for the pathway analyses. Kyoto Encyclopedia of Genes and Genomes (KEGG) were used for pathways analyses.

### Chromosomal diagrams with the locations of differentially methylated regions

The differentially methylated regions from both HIV infected and uninfected twins were plotted in the chromosomal maps by Idiographica V 2.1^13^.

### Virus, cell culture and infection

HIV-1NL4-3 was generated by transfection of plasmid pNL4-3 into HEK 293T cell. The CEM*174 cell line was infected with HIV NL4-3 virus at a MOI = 1. Five days post-infection, CEM*174 T cells were harvested for the further study. Viral replication was monitored by p24 ELISA kit.

### Extraction of genomic DNA, total RNA and protein from cell lines

Genomic DNA, total RNA and proteins from HIV infected and non-infected CEM*174 cell lines were extracted separately. Genomic DNA and total RNA were extracted by QIAamp DNA Mini and QIAamp RNA Blood Mini (QIAGEN) kit separately. Total protein was extracted by lysing the cells in RIPA buffer with protease inhibitor and then centrifuged.

### Determination of relative transcription level by real-time PCR

The mRNA relative expression was determined by real-time PCR with SYBR® Green PCR Master Mix (Applied Biosystems) in triplicate. After normalization with the housekeeping gene ACTB, the relative expression level of the target genes was calculated by ΔΔCt method. Primers used in real-time PCR were listed in Supplementary Table S4

### Western blot analysis

Monoclonal antibodies targeting IGFBP6 (ab135606), SATB2 (ab51502) and DNMT3B (ab13604) were obtained from Abcam and used at 1 μg/ml of 5% non-fat milk with 0.1% Tween-20 (USB). Mouse antibody conjugated with horse-radish peroxidase (HRP) for β-actin was obtained from Santa Cruz (sc-47778) and used at 1:5,000 dilutions. Goat anti-mouse-HRP and Goat anti-rabbit-HRP was obtained from Santa Cruz (sc-2031 and sc-2030) and used at 1:3,000 dilution.

### Bisulfite treatment, methylation-specific PCR (MSP) and bisulfite-sequencing PCR (BSP)

Primers used in the MSP and BSP were listed in Supplementary Table S5. The methylation status of the target genes in the twins and CEM*174 cell lines were determined by bisulfite sequencing using EZ DNA Methylation-Gold™ Kit (Zymo Research) as instructed. The target regions were amplified using Taq polymerase (Invitrogen) cloned into T-vector (Invitrogen) and sequenced as instructions. The resulting sequences of the bisulfite treated DNA were aligned with the reference genome and reported in the form of “lollipop” diagram by BiQ Analyzer^54^. Methylation specific PCR was performed to analyze the methylation status of the target genes in the HIV/AIDS patients and normal subjects. Standard Human WGA Non-Methylated DNA (Zymo Research) were used as positive control in PCR for unmethylated CpGs and negative control in PCR for methylated CpGs; while conversely, Standard Human WGA Methylated (Zymo Research) were used as negative control in PCR for unmethylated CpGs and positive control in PCR for methylated CpGs

### Statistical analyses

Statistical analysis was performed using by SPSS (SPSS, Chicago, IL, USA). Values of *P* < 0.05 were considered statistically significant. The specific statistical tests used were introduced individually. GraphPad Prism5 and Microsoft Excel were used to make the figures.

## Additional Information

**How to cite this article**: Zhang, Y. *et al*. Whole genome methylation array reveals the down-regulation of IGFBP6 and SATB2 by HIV-1. *Sci. Rep*. **5**, 10806; doi: 10.1038/srep10806 (2015).

## Supplementary Material

Supplementary Information

## Figures and Tables

**Table 1:** Annotation analysis of DMRs in CpGi-promoter region from HIV+ twin.

Ontology type	Term	p-Value
**GOTERM_BP_FAT**	regulation of transcription	5.50E-04
	cellular macromolecular complex subunit organization	6.10E-04
	actin polymerization or depolymerization	9.00E-04
**GOTERM_CC_FAT**	non-membrane-bounded organelle	1.20E-08
	intracellular non-membrane-bounded organelle	1.20E-08
	cytosol	3.60E-06
	intracellular organelle lumen	1.20E-05
	membrane-enclosed lumen	1.70E-05
	organelle lumen	3.20E-05
	microtubule cytoskeleton	3.80E-05
	protein-DNA complex	2.10E-04
	ribonucleoprotein complex	7.30E-04
	nuclear lumen	9.20E-04
**GOTERM_MF_FAT**	transition metal ion binding	3.40E-06
	DNA binding	8.20E-06
	zinc ion binding	1.60E-05
**KEGG_PATHWAY**	hsa03010:Ribosome	7.85E-03
	hsa00240:Pyrimidine metabolism	1.66E-02
	hsa00100:Steroid biosynthesis	4.04E-02

*a. Detailed data are shown in Supplemental* Table S3.

*b. Ontology types of BP, CC and MF represents biological process, cellular component and molecular function. KEGG represents KEGG pathway database*.
