# Supplementary material for: Whole genome methylation array reveals the down-regulation of IGFBP6 and SATB2 by HIV-1
Source: Sci Rep. 2015 Jun 3;5:10806. doi: 10.1038/srep10806 (PMC4454074; doi:10.1038/srep10806)
Supplement: Supplementary Information [file srep10806-s1.pdf]

Supplement Information

Title: Whole genome methylation array reveals the down-regulation of *IGFBP6* and *SATB2* by HIV-1

Authors: Yinfeng Zhang[1], Sai-Kam Li[1], Kevin Yi Yang[1, 2], Minghua Liu[1, 2], Nelson Lee[6], Xian Tang[7], Hui Wang[7], Li Liu[3], Zhiwei Chen[3], Chiyu Zhang[4], Jianhua Wang[4, *], Stephen Kwok-Wing Tsui[1, 2, 5,*]

Affiliations:

1 School of Biomedical Sciences, The Chinese University of Hong Kong, Hong Kong.

2 Hong Kong Bioinformatics Center, The Chinese University of Hong Kong, Hong Kong.

3 AIDS Institute, The University of Hong Kong, Hong Kong.

4 Institute Pasteur of Shanghai, Chinese Academy of Sciences, Shanghai, China.

5 Centre for Microbial Genomics and Proteomics, The Chinese University of Hong Kong, Hong Kong.

6 Division of Infectious Diseases, Department of Medicine and Therapeutics, Prince of Wales Hospital, The Chinese University of Hong Kong.

7 HKU-AIDS Institute Shenzhen Research Laboratory and AIDS Clinical Research Laboratory, Shenzhen Key Laboratory of Infection and Immunity, Shenzhen Third People's Hospital, Shenzhen, China.

Correspondence footnote:

* To whom correspondence should be addressed.

Dr. Stephen Kwok-Wing Tsui; E-mail: kwtsui@cuhk.edu.hk; tel. +852-39436381; fax +852-26035123.

Dr. Jianhua Wang; Email: jh_wang@sibs.ac.cn; tel.: +021-63848688; fax: +021-63848688

a

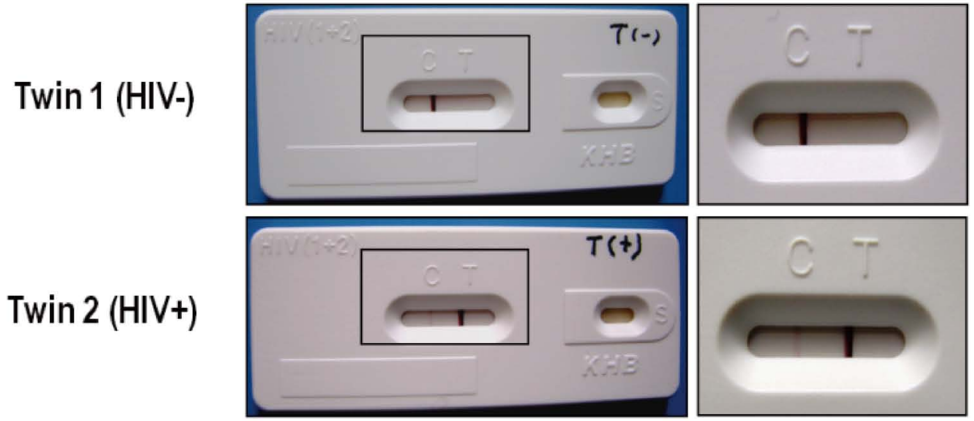

b

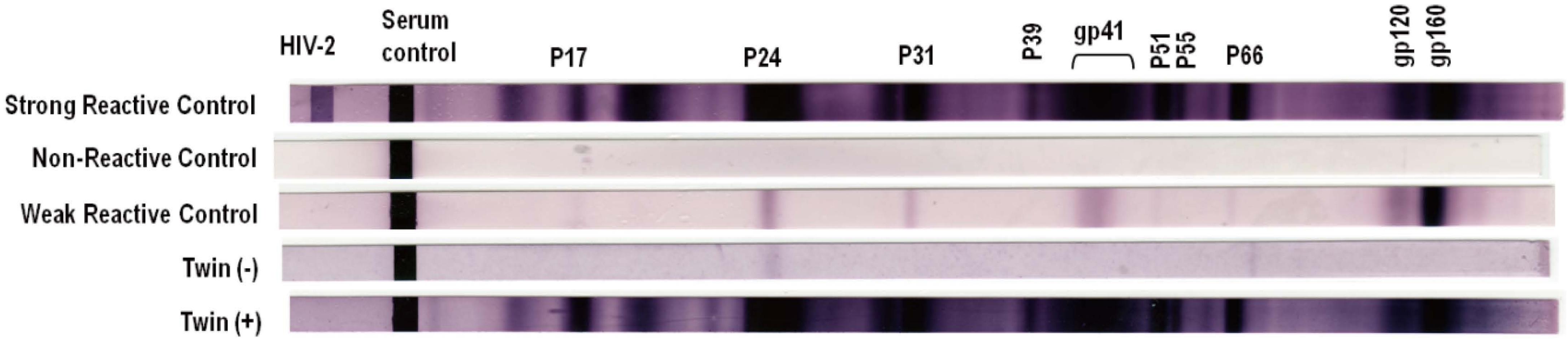

Supplementary Figure S1 | Detection of HIV infection in monozygotic twins. (a) The initial fast test showed that one of the twins was infected with HIV while the other was not. (b) Western blot confirmation tests for HIV proteins in the twin samples. The test serum sample was compared to the strong positive control, weakly positive control and negative controls.

Supplementary Table S1 | Cell enumeration results of the monozygotic twins.

**Subject**	**CD4 /µl**	**CD8 /µl**	**CD3 /µl**	**CD4/CD8**
**Twin (HIV-)**	665	945	1778	0.7
**Twin (HIV+)**	336	1054	1509	0.32
**Reference range in Beijing**	368–1,632	201–931	711–2,353	0.63–3.49
**Reference range in Hong Kong**	396–1,309	224–1,014	723–2271	0.71–2.82
**Reference range in Shanghai**	415-1,189	336-780	NA[a]	0.72-2.56

[a] NA, not applicable

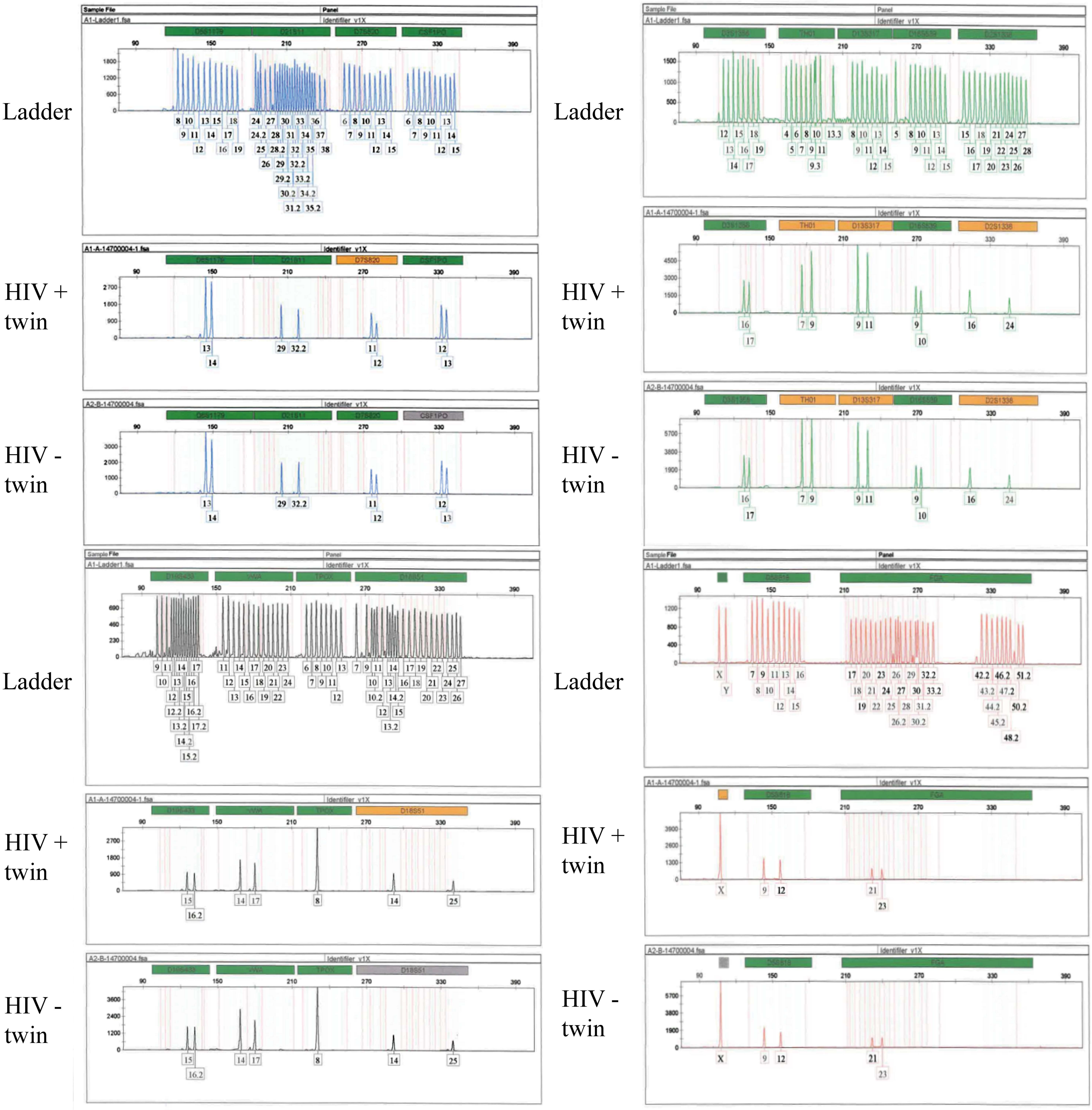

Supplementary Figure S2 | Identification of the monozygotic twins. Short tandem repeats typing of the monozytotic twins in 15 loci and the gender identification marker (amelogenin).

Supplementary table S2 | Genotyping Results of the monozygotic twins

	HIV+ twin		HIV- twin	
Sample ID	[A-14700004]		[B-14700004]	
Locus	Allele		Allele	
D8S1179	13	14	13	14
D21S11	29	32.2	29	32.2
D7S820	11	12	11	12
CSF1PO	12	13	12	13
D3S1358	16	17	16	17
TH01	7	9	7	9
D13S317	9	11	9	11
D16S539	9	10	9	10
D2S1338	16	24	16	24
D19S433	15	16.2	15	16.2
vWA	14	17	14	17
TPOX	8	8	8	8
D18S51	14	25	14	25
D5S818	9	12	9	12
FGA	21	23	21	23
Amelogenin	X,X		X,X	
Male/Female[a]	Female		Female	

The number(s) inside each box are the identified allele(s) for that particular locus

[a] Gender as inferred from the amelogenin typing (XY = male; XX = female)

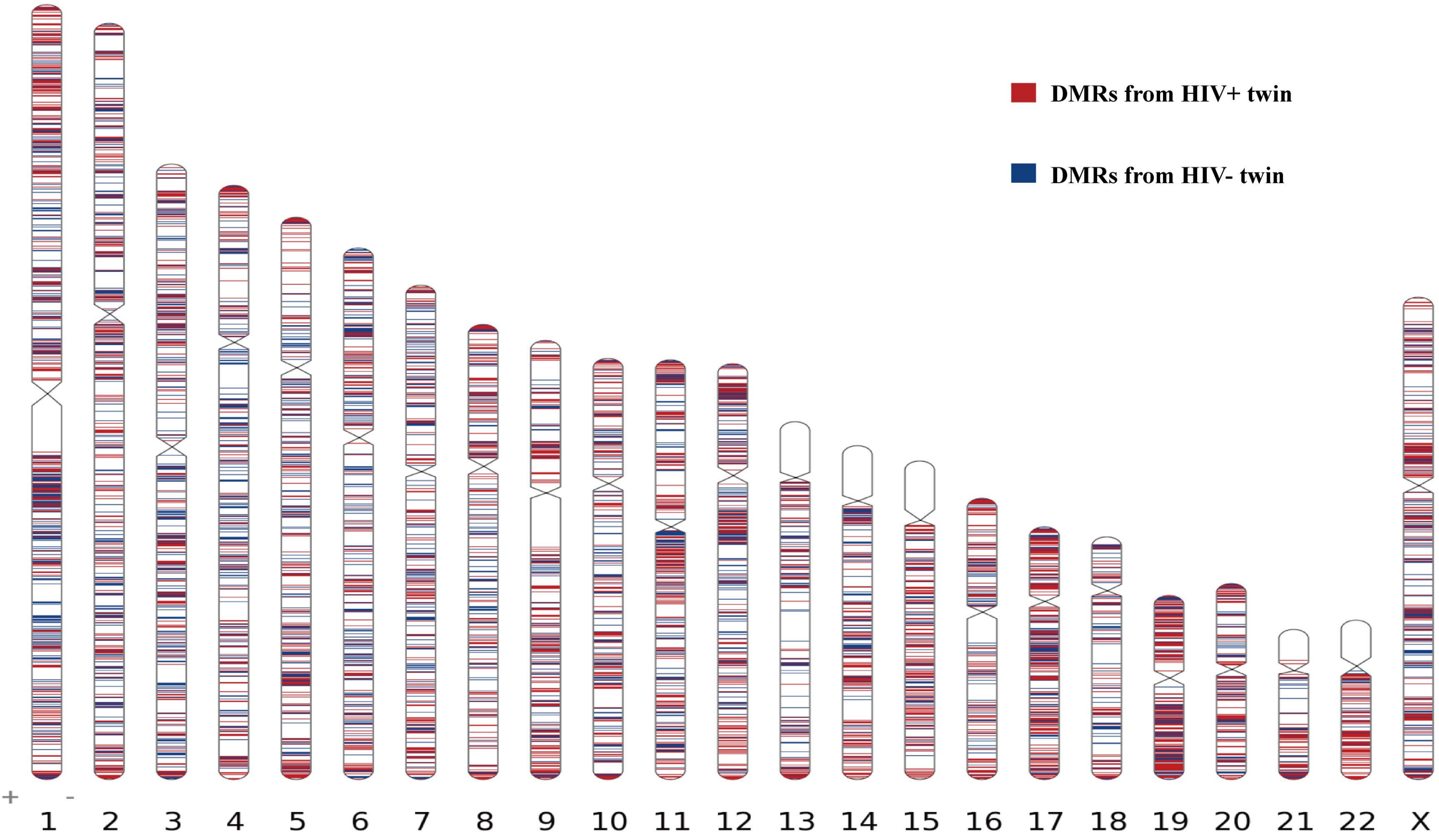

Supplementary Figure S3 | The chromosomal locations of differentially methylated regions in both twins. The red line indicated the DMRs from HIV+ twin while the blue line indicated the DRMs from HIV- twin with the peak value above 3.0.

Supplementary Table S3 | All significant GO-Terms generated by DAVID in different regions in the twins

Supplementary Table S3.1: Gene Ontology categories of the hypermethylated DMRs in CpGi-promoter in HIV+ twin (p<0.01)

Category	Term	Genes Count	P-Value	Benjamini	-log10 (P-Value)
GOTERM_BP_FAT	regulation of transcription	279	5.50E-04	8.40E-01	3.259637311
GOTERM_BP_FAT	cellular macromolecular complex subunit organization	52	6.10E-04	6.30E-01	3.214670165
GOTERM_BP_FAT	actin polymerization or depolymerization	8	9.00E-04	6.30E-01	3.045757491
GOTERM_BP_FAT	transcription	228	1.20E-03	6.20E-01	2.920818754
GOTERM_BP_FAT	translation	47	1.90E-03	7.20E-01	2.721246399
GOTERM_BP_FAT	vacuole organization	11	2.30E-03	7.20E-01	2.638272164
GOTERM_BP_FAT	protein catabolic process	78	2.30E-03	6.70E-01	2.638272164
GOTERM_BP_FAT	cellular macromolecular complex assembly	45	2.60E-03	6.60E-01	2.585026652
GOTERM_BP_FAT	macromolecule catabolic process	94	2.90E-03	6.60E-01	2.537602002
GOTERM_BP_FAT	proteolysis involved in cellular protein catabolic process	75	3.10E-03	6.40E-01	2.508638306
GOTERM_BP_FAT	cellular protein catabolic process	75	3.50E-03	6.50E-01	2.455931956
GOTERM_BP_FAT	protein polymerization	12	3.60E-03	6.20E-01	2.443697499
GOTERM_BP_FAT	DNA packaging	21	3.70E-03	6.10E-01	2.431798276
GOTERM_BP_FAT	cellular macromolecule catabolic process	87	4.50E-03	6.50E-01	2.346787486
GOTERM_BP_FAT	chromatin assembly or disassembly	22	4.50E-03	6.30E-01	2.346787486
GOTERM_BP_FAT	induction of apoptosis by extracellular signals	20	5.10E-03	6.50E-01	2.292429824
GOTERM_BP_FAT	modification-dependent macromolecule catabolic process	71	5.20E-03	6.30E-01	2.283996656
GOTERM_BP_FAT	modification-dependent protein catabolic process	71	5.20E-03	6.30E-01	2.283996656

GOTERM_BP_FAT	macromolecular complex subunit organization	85	5.30E-03	6.20E-01	2.27572413
GOTERM_BP_FAT	lysosome organization	8	5.30E-03	6.00E-01	2.27572413
GOTERM_BP_FAT	protein transport	90	5.90E-03	6.20E-01	2.229147988
GOTERM_BP_FAT	protein maturation	21	6.10E-03	6.10E-01	2.214670165
GOTERM_BP_FAT	long-term strengthening of neuromuscular junction	4	6.30E-03	6.10E-01	2.200659451
GOTERM_BP_FAT	DNA metabolic process	63	7.40E-03	6.60E-01	2.13076828
GOTERM_BP_FAT	nucleosome assembly	16	7.50E-03	6.40E-01	2.124938737
GOTERM_BP_FAT	establishment of protein localization	90	7.50E-03	6.30E-01	2.124938737
GOTERM_BP_FAT	nucleosome organization	17	8.40E-03	6.60E-01	2.075720714
GOTERM_BP_FAT	regulation of transcription, DNA-dependent	188	9.40E-03	6.90E-01	2.026872146
GOTERM_BP_FAT	protein ubiquitination	20	9.70E-03	6.80E-01	2.013228266
GOTERM_CC_FAT	non-membrane-bounded organelle	290	1.20E-08	6.50E-06	7.920818754
GOTERM_CC_FAT	intracellular non-membrane-bounded organelle	290	1.20E-08	6.50E-06	7.920818754
GOTERM_CC_FAT	cytosol	157	3.60E-06	9.80E-04	5.443697499
GOTERM_CC_FAT	intracellular organelle lumen	197	1.20E-05	2.10E-03	4.920818754
GOTERM_CC_FAT	membrane-enclosed lumen	203	1.70E-05	2.30E-03	4.769551079
GOTERM_CC_FAT	organelle lumen	198	3.20E-05	3.40E-03	4.494850022
GOTERM_CC_FAT	microtubule cytoskeleton	74	3.80E-05	3.40E-03	4.420216403
GOTERM_CC_FAT	protein-DNA complex	19	2.10E-04	1.60E-02	3.677780705
GOTERM_CC_FAT	ribonucleoprotein complex	65	7.30E-04	4.80E-02	3.13667714
GOTERM_CC_FAT	nuclear lumen	154	9.20E-04	5.30E-02	3.036212173

GOTERM_CC_FAT	microtubule organizing center	37	1.00E-03	5.40E-02	3
GOTERM_CC_FAT	chromosomal part	51	1.10E-03	5.30E-02	2.958607315
GOTERM_CC_FAT	nucleosome	14	1.80E-03	7.60E-02	2.744727495
GOTERM_CC_FAT	ribosome	32	1.80E-03	7.20E-02	2.744727495
GOTERM_CC_FAT	microtubule organizing center part	13	1.90E-03	6.90E-02	2.721246399
GOTERM_CC_FAT	cytoskeleton	145	2.10E-03	7.10E-02	2.677780705
GOTERM_CC_FAT	microtubule basal body	10	2.20E-03	7.20E-02	2.657577319
GOTERM_CC_FAT	chromosome	57	2.50E-03	7.50E-02	2.602059991
GOTERM_CC_FAT	mitochondrion	117	2.70E-03	7.70E-02	2.568636236
GOTERM_CC_FAT	cytoskeletal part	103	4.40E-03	1.20E-01	2.356547324
GOTERM_CC_FAT	ribosomal subunit	21	4.40E-03	1.10E-01	2.356547324
GOTERM_CC_FAT	chromatin	29	4.50E-03	1.10E-01	2.346787486
GOTERM_CC_FAT	nucleoplasm	96	5.20E-03	1.20E-01	2.283996656
GOTERM_CC_FAT	centrosome	31	6.30E-03	1.40E-01	2.200659451
GOTERM_CC_FAT	gamma-tubulin complex	5	9.80E-03	2.00E-01	2.008773924
GOTERM_MF_FAT	transition metal ion binding	306	0.0000034	0.0038	5.468521083
GOTERM_MF_FAT	DNA binding	260	0.0000082	0.0046	5.086186148
GOTERM_MF_FAT	zinc ion binding	256	0.000016	0.0061	4.795880017
GOTERM_MF_FAT	structural constituent of ribosome	28	0.0014	0.33	2.853871964
GOTERM_MF_FAT	cation binding	410	0.0022	0.39	2.657577319
GOTERM_MF_FAT	metal ion binding	405	0.0029	0.42	2.537602002
GOTERM_MF_F	ion binding	414	0.003	0.38	2.5228787

AT					45
GOTERM_MF_FAT	acid-amino acid ligase activity	30	0.005	0.5	2.301029996
GOTERM_MF_FAT	ligase activity, forming carbon-nitrogen bonds	33	0.0061	0.53	2.214670165
GOTERM_MF_FAT	manganese ion binding	24	0.0077	0.58	2.113509275

Supplementary Table S3.2: Gene Ontology categories of the hypermethylated DMRs in CpGi-promoter in HIV- twin (p<0.01)

Category	Term	Genes Count	P-Value	Benjamini	-log10 (P-Value)
GOTERM_BP_FAT	regulation of neuron apoptosis	11	4.20E-04	0.63	3.37675071
GOTERM_BP_FAT	positive regulation of cell proliferation	27	4.50E-04	0.41	3.346787486
GOTERM_BP_FAT	cell migration	20	8.80E-04	0.49	3.055517328
GOTERM_BP_FAT	synaptic transmission	20	2.10E-03	0.71	2.677780705
GOTERM_BP_FAT	localization of cell	20	3.00E-03	0.75	2.522878745
GOTERM_BP_FAT	cell motility	20	3.00E-03	0.75	2.522878745
GOTERM_BP_FAT	neuron migration	8	3.30E-03	0.73	2.48148606
GOTERM_BP_FAT	negative regulation of neuron apoptosis	7	4.50E-03	0.78	2.346787486
GOTERM_BP_FAT	transmission of nerve impulse	21	5.70E-03	0.81	2.244125144
GOTERM_BP_FAT	regulation of cell proliferation	38	6.40E-03	0.81	2.193820026
GOTERM_BP_FAT	multicellular organismal response to stress	6	6.50E-03	0.78	2.187086643
GOTERM_BP_FAT	negative regulation of programmed cell death	21	7.50E-03	0.8	2.124938737
GOTERM_BP_FAT	transcription	84	7.70E-03	0.78	2.113509275
GOTERM_BP_FAT	negative regulation of cell death	21	7.80E-03	0.75	2.107905397

GOTERM_CC_FAT	synapse part	18	6.30E-04	2.00E-01	3.200659451
GOTERM_CC_FAT	postsynaptic membrane	11	4.90E-03	5.80E-01	2.30980392
GOTERM_CC_FAT	cytosol	54	6.20E-03	5.20E-01	2.207608311
GOTERM_CC_FAT	membrane fraction	36	7.80E-03	5.00E-01	2.107905397
GOTERM_CC_FAT	perikaryon	5	9.60E-03	5.00E-01	2.017728767
GOTERM_MF_FAT	ligand-dependent nuclear receptor activity	8	0.0019	0.72	2.721246399

Supplementary Table S3.3: Gene Ontology categories of the hypermethylated DMRs in non CpGi-promoter in HIV+ twin (p<0.01)

Category	Term	Genes Count	P-Value	Benjamini	-log10 (P-Value)
GOTERM_BP_FAT	sensory perception of chemical stimulus	62	5.8E-15	1.4E-11	14.23657201
GOTERM_BP_FAT	G-protein coupled receptor protein signaling pathway	105	6.3E-15	7.4E-12	14.20065945
GOTERM_BP_FAT	sensory perception of smell	56	1.4E-13	1.1E-10	12.85387196
GOTERM_BP_FAT	cell surface receptor linked signal transduction	143	2.1E-13	1.2E-10	12.67778071
GOTERM_BP_FAT	sensory perception	74	4.3E-10	0.0000002	9.366531544
GOTERM_BP_FAT	cognition	78	2.3E-09	0.0000009	8.638272164
GOTERM_BP_FAT	neurological system process	92	2.7E-08	0.0000089	7.568636236
GOTERM_BP_FAT	defense response	46	0.00021	0.061	3.677780705
GOTERM_BP_FAT	immune response	50	0.00023	0.058	3.638272164
GOTERM_BP_FAT	cellular defense response	10	0.00096	0.2	3.017728767
GOTERM_BP_FA	coagulation	13	0.0012	0.22	2.92081875

T					4
GOTERM_BP_FAT	blood coagulation	13	0.0012	0.22	2.920818754
GOTERM_BP_FAT	polyol transport	4	0.0014	0.23	2.853871964
GOTERM_BP_FAT	hemostasis	13	0.0019	0.3	2.721246399
GOTERM_BP_FAT	positive regulation of interleukin-1 production	5	0.0048	0.55	2.318758763
GOTERM_BP_FAT	cell activation	23	0.0051	0.55	2.292429824
GOTERM_BP_FAT	inflammatory response	25	0.0056	0.56	2.251811973
GOTERM_BP_FAT	response to wounding	36	0.0057	0.55	2.244125144
GOTERM_BP_FAT	acute-phase response	7	0.0062	0.55	2.207608311
GOTERM_BP_FAT	regulation of body fluid levels	14	0.0066	0.56	2.180456064
GOTERM_BP_FAT	regulation of cytokine secretion	6	0.0078	0.6	2.107905397
GOTERM_BP_FAT	adult heart development	4	0.0095	0.66	2.022276395
GOTERM_BP_FAT	positive regulation of interferon-gamma biosynthetic process	4	0.0095	0.66	2.022276395
GOTERM_BP_FAT	regulation of cytokine production	16	0.0096	0.64	2.017728767
GOTERM_CC_FAT	plasma membrane	234	1.20E-08	4.60E-06	7.920818754
GOTERM_CC_FAT	intrinsic to membrane	296	4.10E-05	7.60E-03	4.387216143
GOTERM_CC_FAT	intrinsic to plasma membrane	85	4.90E-05	6.00E-03	4.30980392
GOTERM_CC_FAT	integral to membrane	286	6.60E-05	6.10E-03	4.180456064
GOTERM_CC_FAT	integral to plasma membrane	82	1.10E-04	7.90E-03	3.958607315
GOTERM_CC_FAT	plasma membrane part	130	8.50E-04	5.10E-02	3.070581074
GOTERM_CC_FAT	extracellular region	116	3.80E-03	1.80E-01	2.420216403

GOTERM_MF_FAT	olfactory receptor activity	56	3E-13	2.2E-10	12.52287875
GOTERM_MF_FAT	sugar binding	20	0.00074	0.24	3.13076828
GOTERM_MF_FAT	polyol transmembrane transporter activity	4	0.0014	0.3	2.853871964
GOTERM_MF_FAT	alcohol transmembrane transporter activity	4	0.0014	0.3	2.853871964
GOTERM_MF_FAT	lipid binding	34	0.0019	0.3	2.721246399
GOTERM_MF_FAT	double-stranded RNA binding	7	0.0025	0.31	2.602059991
GOTERM_MF_FAT	cytokine binding	12	0.0071	0.59	2.148741651
GOTERM_MF_FAT	molecular adaptor activity	9	0.0078	0.56	2.107905397
GOTERM_MF_FAT	phospholipid binding	16	0.0094	0.58	2.026872146
GOTERM_MF_FAT	peptide receptor activity	12	0.0099	0.56	2.004364805
GOTERM_MF_FAT	peptide receptor activity, G-protein coupled	12	0.0099	0.56	2.004364805

Supplementary Table S3.4: Gene Ontology categories of the hypermethylated DMRs in non CpGi-promoter in HIV- twin (p<0.01)

Category	Term	Genes Count	P-Value	Benjamini	-log10 (P-Value)
GOTERM_BP_FAT	sensory perception of smell	37	1.2E-13	1.8E-10	12.92081875
GOTERM_BP_FAT	sensory perception of chemical stimulus	38	5.5E-13	4.2E-10	12.25963731
GOTERM_BP_FAT	G-protein coupled receptor protein signaling pathway	59	3.2E-12	1.7E-09	11.49485002
GOTERM_BP_FAT	sensory perception	48	1.1E-11	4.1E-09	10.95860731
GOTERM_BP_FAT	cell surface receptor linked signal transduction	78	2.6E-11	8.1E-09	10.58502665
GOTERM_BP_FAT	cognition	50	5E-11	0.000000013	10.30103
GOTERM_BP_FAT	neurological system process	55	4.6E-09	0.000001	8.337242168

GOTERM_BP_FAT	defense response to bacterium	12	0.000011	0.0022	4.958607315
GOTERM_BP_FAT	response to bacterium	12	0.0014	0.21	2.853871964
GOTERM_BP_FAT	defense response	23	0.0044	0.49	2.356547324
GOTERM_BP_FAT	negative regulation of T cell mediated immunity	3	0.0054	0.53	2.26760624
GOTERM_BP_FAT	negative regulation of lymphocyte mediated immunity	3	0.0074	0.61	2.13076828
GOTERM_BP_FAT	negative regulation of leukocyte mediated immunity	3	0.0074	0.61	2.13076828
GOTERM_CC_FAT	extracellular region	75	4.2E-06	0.00096	5.37675071
GOTERM_CC_FAT	plasma membrane	111	0.0005	0.055	3.301029996
GOTERM_CC_FAT	intrinsic to membrane	147	0.0022	0.15	2.657577319
GOTERM_CC_FAT	intermediate filament	12	0.0026	0.14	2.585026652
GOTERM_CC_FAT	intermediate filament cytoskeleton	12	0.0031	0.13	2.508638306
GOTERM_CC_FAT	integral to membrane	140	0.0059	0.2	2.229147988
GOTERM_CC_FAT	extracellular region part	34	0.0079	0.23	2.102372909
GOTERM_MF_FAT	olfactory receptor activity	38	4.7E-14	2.1E-11	13.32790214
GOTERM_MF_FAT	serine-type endopeptidase inhibitor activity	11	0.000014	0.0032	4.853871964
GOTERM_MF_FAT	endopeptidase inhibitor activity	13	0.000033	0.005	4.48148606
GOTERM_MF_FAT	peptidase inhibitor activity	13	0.000056	0.0063	4.251811973
GOTERM_MF_FAT	enzyme inhibitor activity	15	0.0011	0.092	2.958607315
GOTERM_MF_FAT	anion transmembrane transporter activity	9	0.0093	0.5	2.031517051

Supplementary Table S3.5: Gene Ontology categories of the hypermethylated DMRs in Primary Transcript in HIV+ twin (p<0.01)

Category	Term	Genes Count	P-Value	Benjamini	-log10 (P-Value)
GOTERM_BP_FAT	cellular homeostasis	44	0.00018	0.38	3.744727495
GOTERM_BP_FAT	cell cycle	64	0.00026	0.29	3.585026652
GOTERM_BP_FAT	transcription	142	0.00057	0.39	3.244125144
GOTERM_BP_FAT	cell cycle process	47	0.0016	0.66	2.795880017
GOTERM_BP_FAT	cell cycle phase	36	0.0031	0.81	2.508638306
GOTERM_BP_FAT	cell volume homeostasis	5	0.0048	0.88	2.318758763
GOTERM_BP_FAT	mitotic cell cycle	32	0.006	0.89	2.22184875
GOTERM_BP_FAT	retina development in camera-type eye	7	0.0086	0.94	2.065501549
GOTERM_BP_FAT	ionotropic glutamate receptor signaling pathway	4	0.0093	0.93	2.031517051
GOTERM_BP_FAT	homeostatic process	55	0.0093	0.92	2.031517051
GOTERM_BP_FAT	regulation of neuronal synaptic plasticity	7	0.0099	0.91	2.004364805
GOTERM_CC_FAT	intracellular non-membrane-bounded organelle	175	5.4E-05	0.026	4.26760624
GOTERM_CC_FAT	non-membrane-bounded organelle	175	5.4E-05	0.026	4.26760624
GOTERM_CC_FAT	cytoskeleton	101	0.00022	0.052	3.657577319
GOTERM_CC_FAT	microtubule cytoskeleton	47	0.00074	0.11	3.13076828
GOTERM_CC_FAT	Golgi apparatus	66	0.0014	0.16	2.853871964
GOTERM_CC_FAT	cytoskeletal part	69	0.0033	0.27	2.48148606
GOTERM_CC_FAT	microtubule	26	0.0039	0.27	2.408935393

GOTERM_CC_FAT	N-methyl-D-aspartate selective glutamate receptor complex	4	0.0062	0.35	2.207608311
GOTERM_CC_FAT	cilium part	8	0.0067	0.33	2.173925197
GOTERM_CC_FAT	nuclear lumen	96	0.0071	0.32	2.148741651
GOTERM_CC_FAT	cilium axoneme	6	0.008	0.32	2.096910013
GOTERM_MF_FAT	nucleoside-triphosphatase regulator activity	37	0.0034	0.94	2.468521083
GOTERM_MF_FAT	small GTPase regulator activity	27	0.004	0.81	2.397940009
GOTERM_MF_FAT	GTPase regulator activity	36	0.0042	0.69	2.37675071
GOTERM_MF_FAT	zinc ion binding	152	0.0063	0.74	2.200659451
GOTERM_MF_FAT	chromatin binding	17	0.0074	0.71	2.13076828
GOTERM_MF_FAT	double-stranded RNA binding	7	0.0079	0.67	2.102372909
GOTERM_MF_FAT	Ras guanyl-nucleotide exchange factor activity	12	0.0079	0.61	2.102372909
GOTERM_MF_FAT	RNA binding	55	0.009	0.61	2.045757491

Supplementary Table S3.6: Gene Ontology categories of the hypermethylated DMRs in Primary Transcript in HIV- twin (p<0.01)

Category	Term	Genes Count	P-Value	Benjamini	-log10 (P-Value)
GOTERM_BP_FAT	protein localization	32	0.0018	0.96	2.744727495
GOTERM_CC_FAT	synapse	19	0.00035	0.095	3.455931956

Supplementary Table S4 | All specific primers for real time PCR

Number	Gene	Forward 5`-3`	Reverse 5`-3`
1	ATG12	CACGAACCATCCAAGGACTCA	CCATCACTGCCAAAACACTCAT
2	ATP9B	GGAAGAGGAGTGCGGAAACA	ATGCTCAGCTTGGCTTGAGT
3	BCL2L13	GTTGGCACACAGAAAGCCTG	CCAGCTCCGTTGCTGTCTAA
4	CCDC88A	TCAAAAGGCTGCAACAAGAGAAC	TCGAAGTGCATCTAATTCATCTCG
5	CDNF	GGGCCGACTGTGAAGTATGT	AGCACAGGCGGTTTTCTTTTC
6	EXT1	GCTCTTGTCTCGCCCTTTTGT	TGGTGCAAGCCATTCCTACC
7	EXTL3	CCCGAATGTCACTGGTGGATC	GCTGTCCTTGTTGGAAGCACC
8	FBXW4	GCTATGACACCTATGTTCGCTACTG	GCTCCTCCCACTCCATGACA
9	FEM1B	GCATCTGGCTGTCAACTCC	ATTGTCCACGGCATTCACC
10	FTO	TCTGACCCCCAAAGATGATG	CTCGGAGAATTAGTTTAGGATATTTCA
11	GATAD2A	GACCTCTTCAGCTCGGATGC	TGCTGTGGATTTGCTGAGCC
12	IGFBP6	CCTGCTGTTGCAGAGGAGAA	TCTGTTGGTCTCTGCGGTTC
13	IRAK4	ACAAACCCATAACACCATCAACAT	TGAGGATCAATAAAATCTGACAGCTT
14	MAPRE2	CCACAGCAGGTGCAGCTAAA	TGCTGAGCCACTGGAAGAAG
15	MBD2	ACCCACAACGAATGAATGAACAG	TGGACCAACTCCTTGAAGACCT
16	MCM7	GAATCGTCACTCGTGTCTCTG	TTGGCACTCCTGGCTTGG
17	MYO5A	CAGAGTCCGCTTTATTGATTCC	CCCATGTTCTGACCACTGTAT
18	NAGK	GAGCTGAGGGACCGATTTCC	TGAGCACAACTCCACCATCC
19	PAX5	TCCCAGCTTCCAGTCACAG	GGATGCCGCTGATGGAGTA
20	PURA	AACAAGCGCTTCTACCTGGA	GAGTAAGGCGGCTCTTGTTG
21	RPGRIP1L	GTTAGACGTGAAAGGAGTATC	GTGAGATTGGGTAAAAGCTAG
22	SATB2	GGAGAACGACAGCGAGGAA	CCGATGTATTGCTTTGCCTAGT
23	SLC38A2	CCTATGAAATCTGTACAAAAGATTGG	TTGTGTACCCAATCCAAAACAA
24	SRPK2	CCTTCAGGGTCTGCAGTGAG	TGCAGGCGCTTCTCCAATAA
25	VIPR2	CGTGAACAGCATTCACCCAGAAT	CGTGACGGTCTCTCCCACAT
26	DNMT1	CGACTACATCAAAGGCAGCAACCTG	TGGAGTGGACTTGTGGGTGTTCTC
27	DNMT3A	CGAGTCCAACCCTGTGATGATTG	GCTGGTCTTTGCCCTGCTTTATG
28	DNMT3B	TTGGAATAGGGGACCTCGTGTG	AGAGACCTCGGAGAACTTGCCATC

Supplementary Table S5 | All specific primers for bisulfite sequencing PCR (BSP) and methylation-specific PCR (MSP)

BSP				
Gene	Location of the CpGi	Length of CpGi	Forward 5`-3`	Reverse 5`-3`
SATB2	chr2:200,146,009-200,155,187	9179	ATTTGGTAGAATTGATGATTGTTTG	CCCAACCCCTAACTATAACCTTTAA
IGFBP6	chr12:51,777,840-51,778,222	383	GTTTTAGGAGGGAGGGGTAGGAGTG	TACACCTCAACTTCCTACACTTACC

MSP				
Gene	Type		Forward 5`-3`	Reverse 5`-3`
IGFBP6	Methylated PCR		TGTAGTGTTATTCGTTTAAGGACGAC	TAAAAAACGCGAATCTCACGTA
	Unmethylated PCR		AGTGTTATTTGTTTAAGGATGATGA	ACCTAAAAAACACAAATCTCACATA
SATB2	Methylated PCR		TTGTGTTATTCGGGATTAAGTTATC	TCTATTCCCTTCTCTACTCATAACG
	Unmethylated PCR		TTGTGTTATTTGGGATTAAGTTATTG	TATTCCCTTCTCTACTCATAACACC

Supplementary Table S6.1 | Clinical record of the 8 randomly selected HIV/AIDS patients.

Patient Number	Gender	Age	CD4	Viral load (copies/mL)	Anti-retrovirus therapy
1	Female	56	333	<75	Yes
2	Male	26	55	460000	Yes
3	Male	39	515	<75	Yes
4	Male	40	635	<75	Yes
5	Female	37	246	<75	Yes
6	Male	46	117	2600000	Yes
7	Female	48	135	3700	Yes
8	Male	29	298	<75	Yes

Supplementary Table S6.2 | Clinical record of the 20 age and sex matched HIV/AIDS patients with similar viral load.

Patient Number	Gender	Age	CD4	Viral load (copies/mL)	Anti-retrovirus therapy
1	Male	22	310	6.95×104	No
2	Male	23	311	5.35×103	No
3	Male	23	90	1.21×104	No
4	Male	24	325	1.85×104	No
5	Male	24	322	5.13×104	No
6	Male	27	129	3.83×104	No
7	Male	28	255	6.05×103	No
8	Male	31	374	1.97×105	No
9	Male	31	261	3.08×104	No
10	Male	31	256	1.44×105	No
11	Male	34	263	1.59×105	No
12	Male	34	199	1.09×104	No
13	Male	34	139	1.73×105	No
14	Male	35	336	1.47×105	No
15	Male	36	248	5.64×104	No
16	Male	38	205	3.33×105	No
17	Male	43	387	9.23×104	No
18	Male	46	244	1.81×105	No
19	Male	47	239	2.35×106	No
20	Male	52	243	3.16×104	No

Supplementary Table S6.3 | Clinical record of the 5 HIV/AIDS patients with different viral load.

Subject Number	Sample Date	Gender	Age	CD4	Viral load (copies/mL)
1	20130701	Male	39	270	445000
	20140528			456	<500
2	20130701	Male	27	249	473000
	20140703			660	<500
3	20130702	Male	23	174	314000
	20140620			365	<500
4	20130701	Male	36	29	202000
	20140506			524	<500
5	20130706	Male	29	191	3200000
	20140619			501	<500

Supplementary Table S7.1 | List of 395 overlapped DMRs in HIV+ twin from our study with other reported CD4 + T cell specific DMRs.

AADAC	CBLN2	DZIP3	IQCF1	MYNN	RCAN2	TDRD7
ABCC12	CCDC41	EFEMP1	ITGA2	MYOCD	RNF126	TERF2IP
ABCG1	CCDC92	ENTPD1	KCNMB3	MYOD1	RNF19A	TFB1M
ABLIM1	CD19	ERCC1	KCTD1	NAPA	RNF7	TFDP1
ACBD5	CD53	ERI3	KCTD14	NAV1	RPA3	TGIF1
ACER3	CD9	ERICH1	KIAA0746	NCOR2	RPGRIP1L	TIAM1
ADAR	CDC20	EVL	KIN	NDST4	RPL35A	TIGD1
ADARB1	CDC26	F13A1	KLHDC8A	NDUFC2	RPL6	TJP2
AFF1	CDC42BPB	FADS1	KRT7	NDUFS4	RPS6KA1	TM7SF2
AFF3	CDC7	FAM134B	LEF1	NET1	RPS6KA2	TMEFF2
AGT	CDH2	FAM20A	LILRB1	NFATC1	RPS7	TMEM33
AGTRAP	CECR5	FAM82A1	LOC100133	NIPSNAP1	RPTOR	TNK2
ANGEL2	CEP72	FANCA	LOC285780	NKD1	RPUSD2	TOM1L1
ANKFY1	CFDP1	FBP2	LOC285847	NOVA2	RUFY3	TPD52
ANKRD43	CHAF1B	FBXO22	LOC678655	NPTN	RUNDC2A	TRAF7
AP4M1	CHD1L	FCRLB	LONRF2	NR3C1	RUNX3	TRAM1L1
APBA1	CHD2	FEN1	LRFN3	OASL	SAA2	TRIM71
APBA2	CHD7	FHIT	LRRC2	ODF3B	SAMD10	TTC1
APBB2	CHN2	FLJ43663	LSM1	OPRL1	SCD5	TTC9
APLP2	CKLF	FLT4	LY6D	ORAI2	SCNN1A	TTF2
APOE	CLK1	FOLH1	LY6E	P2RY10	SEPSECS	TTLL8
AQP11	CLK2	FTO	LY86	P4HA2	SERINC3	UEVLD
AQP9	CLSTN3	FXC1	LYSMD4	PAH	SERINC5	UHRF1
ARAF	CNN3	FXN	LYZL4	PAOX	SERPINB10	UNC5D
ARHGAP24	CNO	GALNAC4S	MAGEA10	PCDHB13	SESN1	UNK
ARHGAP9	COL17A1	GALNT6	MAGOH	PCTK1	SFRS5	USP18
ARHGDIB	COL3A1	GAS7	MAN1C1	PDE1B	SFT2D1	UTP14A
ARHGEF3	CPA6	GATA4	MAP2K4	PDE6C	SH3BP4	VAMP1
ATF7	CPE	GCET2	MAP3K7IP1	PDIA3	SIAE	VAV3
ATG12	CPNE7	GLIS1	MAP3K9	PDLIM1	SKIL	VHL
ATP11A	CRTC3	GNAS	MAPT	PEX12	SLAMF7	VPS13A
ATP2B1	CRY2	GNG7	MARK2	PGC	SLC15A4	VPS26B
ATXN1	CRYGA	GNPNAT1	MBD1	PITPNC1	SLC22A18	VWCE
ATXN7	CSGALNACT	GPR18	MBNL2	PKD2L1	SLC37A3	WDR31
AXIN2	CTDSPL	GPR85	MBP	PLAGL1	SLC39A12	WIPI2
AXL	CTNNA1	GRAP2	MCM7	PLD1	SLC5A3	WNT6
B3GNT2	CTSH	GTSE1	MDS2	PLEKHA1	SLC5A6	WTAP
BACH2	CXCR5	GYPB	MEF2B	PLEKHA5	SLFN13	XAF1
BCAS3	CYB561	GYPE	MEP1A	POLR3D	SMAD3	ZAK
BCKDHB	DCHS1	HADH	MEST	POU2F2	SMAP2	ZBED4
BCL2L14	DDIT4	HCCA2	MFN2	PPM1H	SMS	ZBTB26
BRS3	DDX58	HDAC4	MGAT4A	PPP1R7	SMURF2	ZCCHC14
BTBD19	DGKE	HERC5	MGAT5B	PRICKLE1	SNIP1	ZDHHC1
C11orf49	DGKZ	HGF	MGP	PRKCA	SNX29	ZEB2
C17orf105	DHTKD1	HIST1H1D	MIA2	PRMT2	SP1	ZFPL1
C17orf108	DICER1	HIST1H2BH	MKI67	PRR16	STAM	ZFYVE28
C17orf75	DIDO1	HNMT	MLLT3	PSD3	STK39	ZHX2
C2orf85	DLX1	HOXA6	MMP9	PSMB5	SULT1A1	ZNF322B
C6orf203	DMRT1	HOXB4	MORC2	PSPN	SUV420H1	ZNF423
CALR	DNASE2B	HUWE1	MPZL3	PTHLH	SVIL	ZNF559
CAMK2D	DNMT3B	IDI2	MRPL49	PYGM	TARDBP	ZNF622
CASP1	DPM1	IFI44L	MRPS6	RAB24	TBC1D22A	ZNF625
CASP10	DRD1	IFITM3	MSH3	RAB32	TBCD	ZSCAN1
CAV3	DUSP3	IL4R	MTRR	RAB4A	TBX3	
CBFA2T3	DUSP6	INPP5F	MTSS1	RASSF5	TC2N	
CBL	DYRK2	IQCD	MYH10	RB1CC1	TCTEX1D4	

Supplementary Table S7.2 | List of 206 overlapped DMRs in HIV- twin from our study with other reported CD4 + T cell specific DMRs.

ABL2	CBR4	ETS1	IFI44	NLRC5	RGS12	STK24
ACACA	CCM2	ETV5	IL1RL1	NMS	RIPK2	TAGAP
ACLY	CD47	FAM107B	INSIG1	NOS1AP	RNF213	TAS2R60
ACSF3	CD8A	FAM38B	IREB2	NOVA1	RYR2	TEKT3
ACTA2	CDH11	FAM65B	ITGB1	NR4A2	SAMD4A	TEX2
AHR	CDK6	FBXO18	JAK1	NT5DC1	SBNO2	TGFBR1
ALKBH6	CDS2	FGF11	KBTBD4	NUP155	SCOC	THAP2
ARHGAP25	CKAP5	FNDC3A	KCNK13	OSBPL3	SCRN1	TLR6
ARID1B	CLOCK	FOXP1	KIAA1026	PAQR7	SCYE1	TNFRSF19
ARID5B	CNTF	FOXP3	KIAA1324L	PARN	SEC24C	TNFSF11
ARIH1	CRB1	FSTL1	KIAA1462	PAX9	SEMA4C	TOR1AIP2
ARPP-21	CREG1	FUT8	KIRREL	PCDHA9	SERPINB12	TP53BP1
ATF7IP	CTNNA2	GALNT3	KLF13	PDCD10	SERTAD4	TRRAP
AUTS2	CYP27B1	GFI1	KLHL13	PDE3B	SFRS12	TSPAN5
AZU1	DCTN3	GGH	KRT10	PGM5	SGK269	TXNDC9
BCAT1	DDX27	GLDN	KRT2	PHEX	SGPP1	UBAC2
BCL2	DDX60	GPBP1L1	LCP1	PHF20L1	SGPP2	VGLL4
BIN1	DIP2C	GPR183	LECT2	PPAT	SIRPB1	VPS54
BIN3	DLG1	GPR75	LIFR	PRH1	SLC6A2	XPO1
BRE	DPY19L1	GRIA3	LOC285733	PRSS3	SMAD6	XRN1
C12orf26	EDAR	GRID2IP	LPIN2	PTGFR	SMEK2	ZBTB20
C18orf20	EDARADD	GRM3	MAD1L1	PTPRN2	SNRK	ZCCHC2
C19orf22	EDNRB	GRM5	MAGEE2	RAB11B	SOCS4	ZFP91
C1orf133	EGR4	HCP5	MBNL1	RAB11FIP2	SOX5	ZFP91-CNTF
C8orf44	EHHADH	HIRA	MEF2C	RABGAP1L	SPEG	ZHX1
C9orf93	EIF2AK2	HKR1	MIB1	RAG1AP1	SRCAP	ZNF148
CACNA1D	EML1	HNRPLL	MPRIP	RASSF3	SS18L2	
CAND1	EPDR1	HOXD4	MRPS14	RBM38	SSH1	
CARD8	ESR2	HRAS	NDST3	RERE	ST5	
CASKIN2	ETNK2	HSPA4	NFKB1	REV1	ST6GALNAC	

Supplementary Table S7.3 | List of 142 overlapped DMRs in HIV+ twin from our study with literature reported DMRs between other monozygotic twins.

AATF	CCDC55	GLRX5	LY6D	OR4D10	RSU1	TSGA14
ABL1	COPG2	GLTSCR2	MAP3K7	OXCT2	SART1	TSPAN33
ABO	CREBBP	GP6	MARCKSL1	P2RY1	SC4MOL	TUBA8
ACOT4	CSMD1	GRK1	MBNL2	PDLIM7	SCRT2	TUBB6
ALOX12	DBI	HABP4	MEIS1	PGAM2	SEC23B	VAMP1
AMY1A	DCC	HEBP1	MOBKL3	PKN2	SERPINB3	VHL
ANKRD55	DDAH1	HIST1H2AG	MOV10	PLAGL1	SETDB2	VWCE
AP3S1	DFFB	HIST3H2BB	MPST	PLEKHO1	SLC12A5	WNT6
APPBP2	DLC1	HRASLS	MRP63	PNPT1	SLC22A18	YKT6
ARHGEF11	DRD1	IFITM2	MRPL20	PPP1R2	SMPD3	ZDHHC14
ATP9B	EDIL3	IQCD	MUC4	PPP1R9A	SOX1	ZNF555
ATXN3	ERH	KCND3	MYH7	PRPF38B	SSR2	ZNF577
BCYRN1	ETV1	KIAA0802	MYOD1	PXDN	ST6GALNAC	ZNF622
BMP15	EXT1	KIF3C	NCOR2	RANBP1	TBC1D3	ZNF645
BRCA1	FAIM	KIR3DL2	NEDD1	RARG	TBXAS1	ZNF664
BRD3	FBXO7	KLHDC7A	NEUROG1	RDH11	TESSP1	ZNF83
C14orf39	FCRL3	KLHL22	NKIRAS2	RHCG	TFDP2	
C20orf151	FLJ41484	KPNA5	NOMO1	RNF11	TGFBI	
C3orf31	GADD45G	LAMB1	NSUN5C	RPL23A	THOC3	
C9orf3	GALNT11	LCN1	NUDT11	RPL7A	TRIM60	
CACNG5	GFRA2	LILRB3	OLIG2	RRM1	TRPV1	

Supplementary Table S7.4 | List of 40 overlapped DMRs in HIV- twin from our study with literature reported DMRs between other monozygotic twins.

ALDH18A1	CCDC59	FRZB	ICA1	MYCBP2	PIWIL1	THRB
ALX3	CCND1	GPR22	KIAA1486	MYOM2	PSKH2	TXNDC9
BCL2	CCRL2	GRM3	KIT	NCOA5	RASGRP1	VIPR1
C15orf29	COL21A1	GTF3C3	KREMEN1	NR4A2	RELN	ZNF148
C3orf19	DNMT3L	HEYL	LCP1	NTRK3	SPRY2	
C9orf89	DOCK2	HSPH1	MCM3	PDE11A	SSPN	

Supplementary Figure S4 | The sequencing result from PCR product of MSP.

Unmethylated IGFBP6:

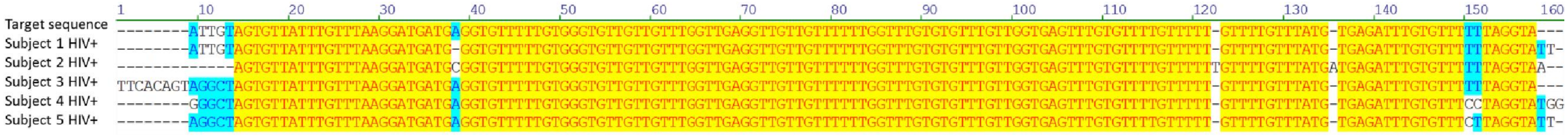

Methylated IGFBP6

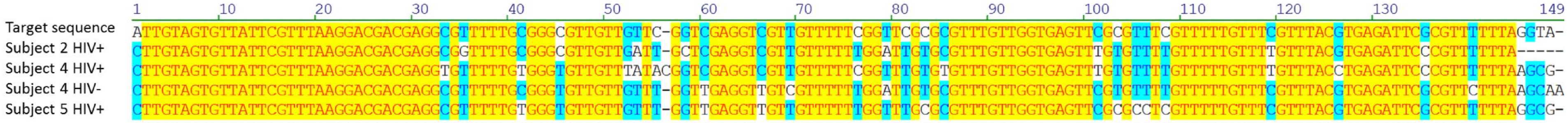

Unmethylated SATB2

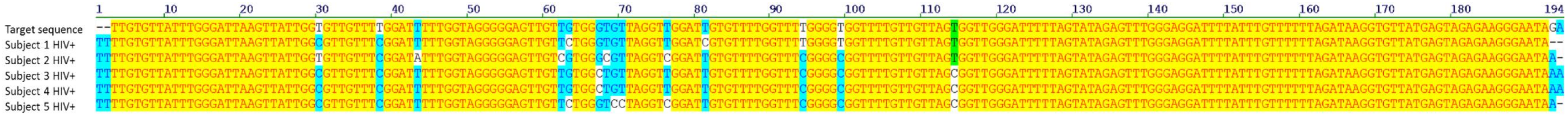

Methylated SATB2 *

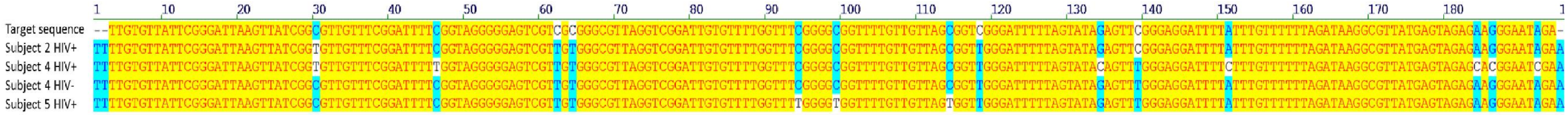

* No sequencing result can be obtained from the very weak band of the sample from Subject 1 HIV- due to low signal.

Full length blotting images for Figure 3a, 3b and Figure 6b

Figure 3a

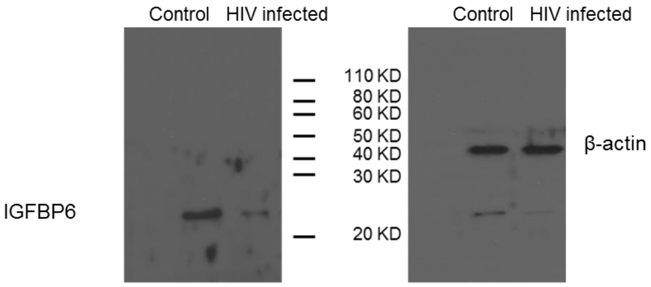

Figure 3b

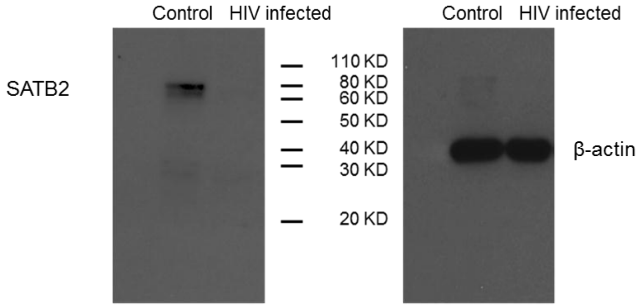

Figure 6b

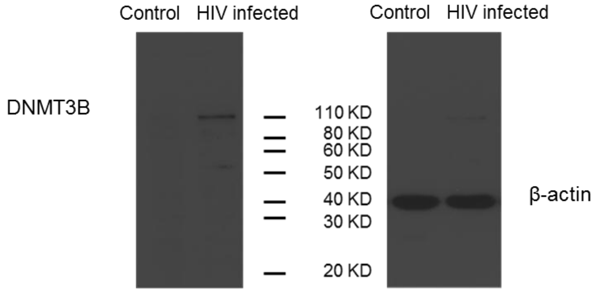

Full unedited gel for Figure 4c, 4d and 5c

Figure 4c

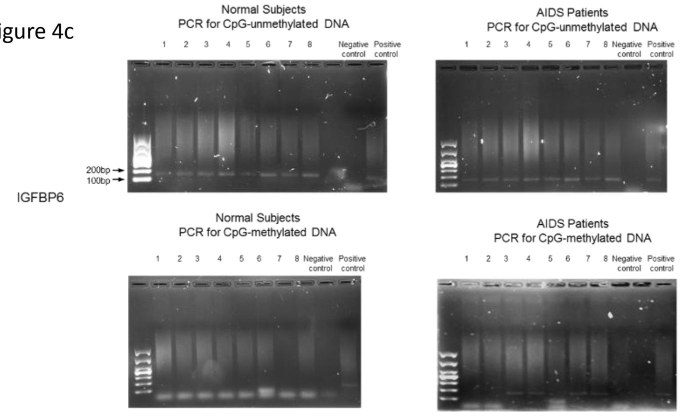

Figure 4d

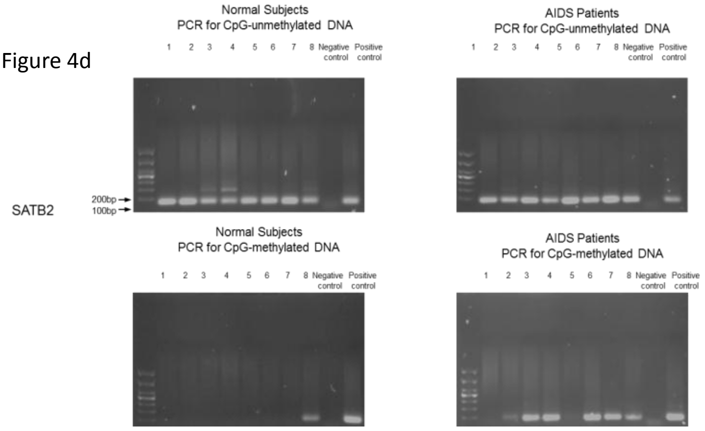

Figure 5c

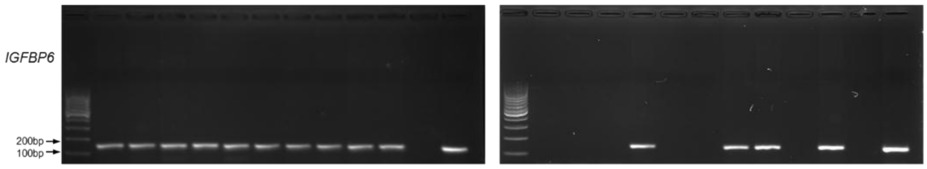

Figure 5d

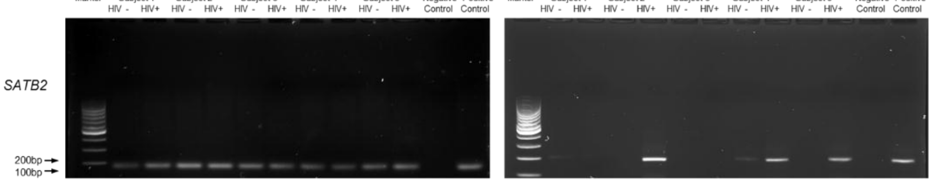

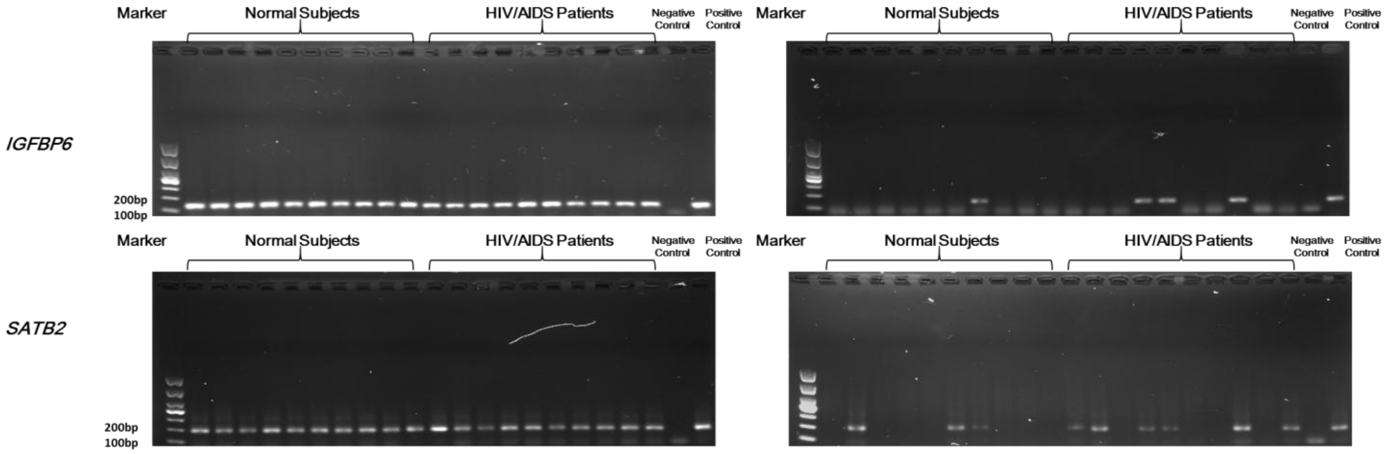

Marker
Normal Subjects
HIV/AIDS Patients
Negative Control
Positive Control
IGFBP6
SATB2
200bp
100bp
